# Is there a neural common factor for visual illusions?

**DOI:** 10.1167/jov.26.10.13

**Published:** 2026-10-13

**Authors:** Maya A. Jastrzębowska, Ayberk Ozkirli, Aline F. Cretenoud, Bogdan Draganski, Michael H. Herzog

**Affiliations:** 1 Laboratory of Psychophysics, Brain Mind Institute, School of Life Sciences, École Polytechnique Fédérale de Lausanne (EPFL), Lausanne, Switzerland; 2 Laboratory for Research in Neuroimaging (LREN), Centre for Research in Neurosciences, Department of Clinical Neurosciences, Lausanne University Hospital and University of Lausanne, Lausanne, Switzerland; 3 Department of Psychology, University of Warsaw, Warsaw, Poland; 4 Neuro-X Institute, School of Engineering, École Polytechnique Fédérale de Lausanne (EPFL), Geneva, Switzerland; 5 Imaging Neuroscience of Ageing – inAGE laboratory, Neurology Department-Inselspital, University Hospital Bern, University Bern, Bern, Switzerland; 6 Institute for Diagnostic and Interventional Neuroradiology, Inselspital Bern, University of Bern, Bern, Switzerland; 7 Laboratory for Research in Neuroimaging (LREN), Centre for Research in Neurosciences, Department of Clinical Neurosciences, Lausanne University Hospital and University of Lausanne, Lausanne, Switzerland

**Keywords:** illusions, population receptive field mapping, interindividual variability, functional magnetic resonance imaging, brain–behavior mapping

## Abstract

It has been reported that the surface area of the human primary visual cortex predicts the susceptibility to several spatial contextual illusions. Yet, contrary to common belief, illusions correlate only weakly at the perceptual level, raising the question of how they can correlate with a neural measure like V1 surface area. In addition, mounting evidence suggests that there is substantial interindividual variability not only in neural function and anatomy but also in the mapping between the two, which further challenges the findings of a neural common factor for illusions. In light of these concerns, our first goal was to re-evaluate the previous imaging results. Seeking to investigate whether these findings generalize, we expanded our study to other illusions. We could not replicate previous findings and did not find any other significant correlations, either at the perceptual level or between illusion susceptibility and visual functional neuroanatomy.

## Introduction

Humans are highly idiosyncratic in their appearance, personality traits, cognitive and perceptual abilities, brain anatomy, and physiology. For example, V1 size can vary more than threefold between individuals (Benson et al., 2022; Dougherty et al., 2003; Stensaas, Eddington, & Dobelle, 1974). It is tempting to relate this large variability in neural measures to variabilities in perception. A series of studies has posited that V1 surface area may predict illusion susceptibility via the involvement of lateral connections in spatial contextual effects (Moutsiana et al., 2016; Schwarzkopf, 2015; Schwarzkopf, Song, & Rees, 2011; Schwarzkopf & Rees, 2013; Song, Schwarzkopf, & Rees, 2013). Specifically, because the length of intracortical projections may be limited by anatomical constraints and do not scale at the same rate as V1 surface area, a larger V1 would coincide with a decreased influence of context (Moutsiana et al., 2016; Schwarzkopf, 2015; Schwarzkopf et al., 2011; Schwarzkopf & Rees, 2013; Song et al., 2013). This hypothesis predicts an inverse relationship between V1 surface area and illusion magnitude. Indeed, Schwarzkopf et al. reported that susceptibility to the Ebbinghaus (EB) and Ponzo “hallway” (PZh) illusions correlates negatively with the surface area of the primary visual cortex V1 (Schwarzkopf et al., 2011; Schwarzkopf & Rees, 2013). Similarly, Song et al. (2013) found a significant negative correlation between V1 surface area and susceptibility in the tilt (TT) illusion (referred to by the authors of that study as the “orientation contextual illusion”) magnitude. Furthermore, Moutsiana et al. (2016) reported a significant correlation between Delboeuf (DB) illusion strength and surface area and population receptive field (pRF) sizes in V1.

The above reports linking V1 surface area to various spatial contextual illusions posit a neural common factor. A common factor for illusions should then be present at the behavioral level, for example, greater susceptibility to the EB illusion should go hand in hand with greater susceptibility to the PZ illusion. Yet this has not been found to be the case across a multitude of studies (Bertamini, Herzog, & Bruno, 2016; Cretenoud et al., 2019; Cretenoud, Francis, & Herzog, 2020; Cretenoud, Grzeczkowski, Bertamini, & Herzog, 2020; Grzeczkowski, Clarke, Francis, Mast, & Herzog, 2017; Mazuz, Kessler, & Ganel, 2023; Schwarzkopf et al., 2011; Takahashi, Ujiie, & Dai, 2023). On the contrary, recent studies have shown that visual illusion magnitudes correlate only weakly with each other, even though the test–retest reliability is usually good (Cretenoud et al., 2019; Cretenoud, Francis, et al., 2020; Cretenoud, Grzeczkowski, et al., 2020; Grzeczkowski et al., 2017). Notably, also in one of the aforementioned neuroimaging studies (Schwarzkopf et al., 2011), V1 surface area was found to correlate with EB and PZ illusion magnitudes but the magnitudes of the two illusions did not significantly correlate with each other (*r* = 0.24, *p* = 0.208). Moreover, the correlations between illusion magnitudes and cortical properties were limited to V1 and not found in higher visual areas, such as V2, V3, or V4 (Moutsiana et al., 2016; Schwarzkopf et al., 2011; Schwarzkopf & Rees, 2013; Song et al., 2013), even though the surface areas of the adjacent areas (e.g., V1 and V2) are known to be highly correlated within individuals (Dougherty et al., 2003).

This multitude of findings is not easy to reconcile. Our aims here were first to re-evaluate the validity of previous findings and second to test a variety of further illusions to systematically study the common neural structure of illusion perception. To this end, we tested a battery of 13 visual illusions, including the Delboeuf, Ebbinghaus, Ponzo “hallway”, and tilt illusions, which were previously shown to correlate with V1 size (Moutsiana et al., 2016; Schwarzkopf et al., 2011; Schwarzkopf & Rees, 2013; Song et al., 2013). As a form of control, we included illusions for which we had no a priori reason to expect a mediating role of V1 size, including the contrast (CS) illusion for which no correlation was reported (Song et al., 2013).

## Methods

### Participants

Thirty paid volunteers (14 females), 18 to 35 years of age (mean, 24.7 ± 4.45 years) participated in the experiment. All but six were right-handed. All participants had normal uncorrected visual acuity—both binocular and monocular in both eyes—as assessed with the Freiburg Visual Acuity test, that is, acuity values of greater than 1.0 (Bach, 1996). The experiment included a psychophysics part—carried out in a behavioral testing room—and a functional magnetic resonance imaging (fMRI) part, which immediately followed the psychophysics part. Participants gave written informed consent and were made aware that they could discontinue the experiment at any time. All experimental procedures complied with the Declaration of Helsinki, except for pre-registration (§35), and were approved by the local ethics committee. All participants were naïve to the purpose of the experiment.

### Stimuli and apparatus

For the psychophysics experiment, visual stimuli were displayed on an ASUS VG278HE LCD monitor (ASUS, Gauteng, South Africa; refresh rate, 60 Hz; spatial resolution, 1,920 × 1,080) at a viewing distance of 76 cm. The mean luminance of the screen was 75 cd/m^2^. Unless otherwise stated, the stimuli were white (150 cd/m^2^) and presented on a black background. Stimulus programs were implemented in MATLAB (The MathWorks, Inc., Natick, MA) using Psychtoolbox (Brainard, 1997).

The MRI experiment was conducted at the MRI-scanning facilities of the Department of clinical neurosciences at the Centre Hospitalier Universitaire Vaudois (CHUV) in Lausanne, Switzerland. Participants lay in the MRI scanner and looked at a screen, placed at the end of the 60-cm scanner bore, through a head-coil–mounted mirror (viewing distance, 70 cm). A Sony VPL-FH31 projector (Sony, Sony City, Japan) size of projected image, 57 × 32.1 cm; chosen pixel resolution, 1,920 × 1,080 pixels; refresh rate, 60 Hz) was used to back-project the stimuli onto the screen, with a mean luminance of 1,000 cd/m^2^. As in the psychophysics part, stimulus generation and response collection were done in MATLAB using Psychtoolbox. All participants completed the MRI session immediately after the behavioral session.

#### Illusion stimuli

Thirteen illusions were tested (Figure 1): the bisection (BS), CS, DB, two variants of the EB (EB1 and EB2), extinction (EX), honeycomb (HC), Müller–Lyer (ML), Poggendorff (PD), two variants of the PZ illusion (PZ and PZh), TT, and Zöllner (ZN) illusions. Each illusion was tested with two reference-dependent conditions; for example, participants were asked to adjust the length of the vertical segment to match the length of the horizontal segment in the BS illusion, or vice versa. Illusions were tested using an adjustment procedure, with participants performing the adjustment with a computer mouse and right clicking to validate the trial. Each condition was tested twice, making up 52 trials in total (13 illusions × 2 reference-dependent conditions × 2 trials), presented in a random order. No feedback was provided and there was no time limit, that is, the stimuli were shown until a response was given. Unless otherwise specified, a free viewing condition was used; that is, there was no fixation point. Lines were shown with a 4-pixel width, except in the DB illusion, where the circles were 2 pixels wide.

**Figure 1.**
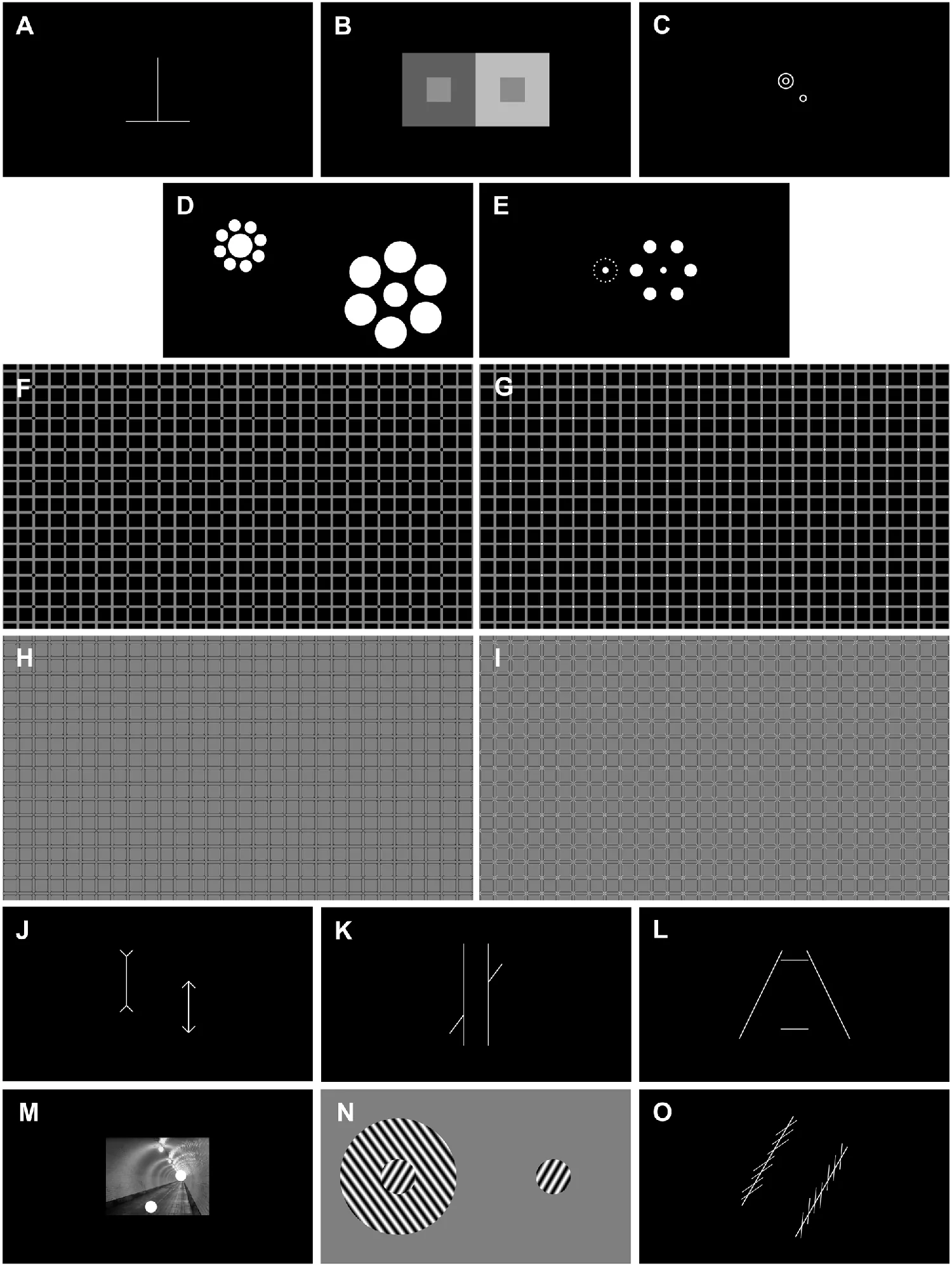
The 13 illusions tested. (**A**) The BS illusion. Participants were instructed to adjust the length of the horizontal segment to match the vertical segment or vice versa. (**B**) The CS illusion. Participants were instructed to adjust the shade of gray of the left inner square to match the right inner square or vice versa. (**C**) The DB illusion, similarly to previously referenced work (Moutsiana et al., 2016). Participants were instructed to adjust the size of the inner top-left circle to match the bottom-right circle or vice versa. (**D**, **E**) Two variants of the EB illusion (EB1 and EB2, respectively). EB2 as in previously referenced work (Schwarzkopf & Rees, 2013; Song et al., 2013). Participants were instructed to adjust the diameter of the left central disk to match the right central disk or vice versa. (**F**, **G**) The EX illusion with black and white dots, respectively. While fixating the center of the screen, participants were instructed to adjust the size of the centered red ellipse—not shown here—so that all dots perceived in the periphery of the uniform texture lie within it. (**H**, **I**) The HC illusion with black and white barbs, respectively. While fixating the center of the screen, participants were instructed to adjust the size of the centered red ellipse—not shown here—so that all barbs perceived in the periphery of the uniform texture lie within it. (**J**) The ML illusion. Participants were instructed to adjust the length of the left shaft to match the right shaft or vice versa. (**K**) The PD illusion. Participants were instructed to adjust the vertical position of the left/right interrupted diagonal so that it lies on a continuum with the right/left interrupted part, respectively. (**L**) The PZ illusion. Participants were instructed to adjust the length of the upper/lower horizontal segment to match the lower/upper horizontal segment, respectively. (**M**) The PZh or corridor illusion, as in previously referenced work (Schwarzkopf et al., 2011). Participants were instructed to adjust the size of the lower-left disk so that it matches the upper-right disk or vice versa. (**N**) The TT illusion, similarly to previously referenced work (Song et al., 2013). Participants were instructed to adjust the orientation of the right disk to match the left inside disk or vice versa. (**O**) The ZN illusion. Participants were instructed to adjust the orientation of one main stream so that it appears parallel to the other main stream) illusions. Illusions (**F**) to (**I**) were presented such that they covered a large proportion of the visual field (Bertamini et al., 2016). All illusions except the EX and HC illusions [(**F**) to (**I**)] were tested with two configurations; for example, in the PZ illusion, either the upper horizontal line was adjusted to match the length of the lower horizontal line or vice versa.

##### Bisection (BS) illusion (also called vertical–horizontal)

Participants had to adjust the length of the vertical segment so that it appeared to be the same length as the horizontal segment or to adjust the length of the horizontal segment to match the length of the vertical one (Figure 1A). The reference segment was 10.4° long and the adjustable segment had a size randomly set in the range of 2° to 17° at the beginning of each trial. The horizontal segment was displayed 5.2° to the bottom of the middle of the screen and was always touching the vertical segment.

##### Contrast (CS) illusion

We asked participants to adjust the shade of gray of the left inside square so that they perceived it to be the same as the shade of gray of the right inside square, or vice versa (Figure 1B). The luminance values of the dark gray outside square, light gray outside square, and reference square were 17.46 cd/m^2^, 75.06 cd/m^2^, and 38.58 cd/m^2^, respectively. The sides of the inside squares were 4° in length. The inside squares were displayed in the middle of two outside squares, whose sides were 12° in length. The entire inside–outside square configuration was displayed in the middle of the screen.

##### Delboeuf (DB) illusion

The stimulus was similar to that in previously referenced work (Moutsiana et al., 2016), with the distinction that in that case, the authors used a multiple alternative perceptual search procedure, in which participants had to report which of four comparison rings was more similar in size to a central target. Here, we used an adjustment procedure in which participants had to adjust the size of the upper-left inside circle to match the size of the lower-right circle or vice versa (Figure 1C). The reference and upper-left outside circles were 0.98° and 2.35° in diameter, respectively. The size of the adjustable circle was randomly set at the beginning of each trial, but it never exceeded 2.35°. The reference and adjustable circles were 3.92° from each other and the whole illusion was centered in the middle of the screen.

##### Ebbinghaus (EB1 and EB2) illusions

In both variants of the EB illusion, participants were instructed to adjust the size of the left central disk (i.e., left target) to match the size of the right central disk (i.e., right target), or vice versa.

We measured the susceptibility to the first variant of the EB illusion (EB1) illusion as we did previously (Cretenoud et al., 2019). The left and right targets were surrounded by eight small (2.0° in diameter) and six large (5.2° in diameter) flankers, respectively (Figure 1D). The reference target was 4° in diameter. The centers of the small and large flankers were 3.36° and 6.08° away compared with the center of the left and right targets, respectively. The left and right targets were centered 12.41° to the left and right and 4.00° to the top and bottom, respectively, compared with the center of the screen.

The second variant of the EB illusion (EB2) was strongly inspired by previously referenced work (Schwarzkopf & Rees, 2013), that is, the stimulus dimensions matched those described in that work though the luminance conditions were different. It should be noted that the authors of the referenced article have since made known in a comment to that work that there was an error in the stimulus description, with the radii of the stimuli mistakenly referred to as the diameters (Schwarzkopf & Rees, 2013). Our stimulus was designed based on the original description; that is, the diameters in our stimulus correspond with one-half of the diameters of the stimuli used in the original study (Schwarzkopf & Rees, 2013). Our stimulus dimensions were as follows: the left and right targets were surrounded by 16 small (0.26° in diameter) and six large (2.07° in diameter) flankers, respectively (Figure 1E). The small and large flankers were located 1.86° and 4.34° away from the center of the left and right targets, respectively. The reference target was 1.03° in diameter. The left and right targets were located at 4.65° eccentricity compared with the middle of the screen and were vertically centered.

In both variants of the EB illusion, the size of the adjustable target was randomly chosen by the computer at the beginning of each trial with the constraint that it could never touch the flankers.

##### Extinction (EX) and Honeycomb (HC) illusions

The stimuli used here were the same as in previously referenced work (Bertamini et al., 2016; Bertamini, Cretenoud, & Herzog, 2019). In the HC and EX illusions, participants are unable to see shapes (barbs in the HC illusion and dots in the case of the EX illusion) in the periphery of a uniform texture, while they are fixating the center of the texture.

Participants were asked to fixate a red central cross while adjusting the size of a red ellipse on both *x* and *y* dimensions, so that they could perceive all barbs (HC illusion) and dots (EX illusion) inside the ellipse. The initial size of the red ellipse was randomly chosen at the beginning of each trial, with the screen size as limits. Two contrast polarity conditions, that is, black or white barbs (HC illusion) or dots (EX illusion), were tested for each illusion, making up four conditions (Figures 1F–I). To reduce the aftereffect following a HC or EX trial, 30 random light and dark gray checkerboards made of squares (0.52° length of each side) with 0.35 and 0.65 of the maximum luminance were presented for 0.5 second each.

##### Müller-Lyer (ML) illusion

Participants had to adjust the length of the left shaft (i.e., the vertical segment with inward-pointing arrows) so that they perceived it to be as long as the right shaft (i.e., the vertical segment with outward-pointing arrows), or vice versa (Figure 1J). The reference shaft was 8.0° long and the fins were 1.5° long, oriented at 45.0° compared with the vertical. At the beginning of each trial, the length of the adjustable shaft was randomly set between 2° and 21°. The left and right shafts were centered 2.12° to the top and bottom and 4.97° to the left and right compared with the middle of the screen, respectively.

##### Poggendorff (PD) illusion

Participants were instructed to adjust the vertical position of the left or right interrupted diagonal so that it appeared to lie on a continuum with the right or left interrupted part, respectively (Figure 1K). The two main vertical streams were 16.6° long and 4.0° away from each other. Both parts of the interrupted diagonal were 3.8° long and titled by 37.0° compared with the vertical. When the position of the left part of the interrupted diagonal was adjusted, the right part was touching the right main stream 6.29° away compared with the top of the main streams. When adjusting the position of the right part of the interrupted diagonal, the left part touched the left main stream 5° away compared with the bottom of the main streams. The adjustable element was randomly positioned along the corresponding main stream at the beginning of each trial and the whole illusion was centrally displayed.

##### Ponzo (PZ) and Ponzo “hallway” (PZh) illusions

In the first variant of the PZ illusion, the task was to adjust the length of the upper or lower horizontal segment to match the length of the lower or upper horizontal segment, respectively (Figure 1L). The reference segment was 4.5° long, while the length of the adjustable segment was randomly set between 0.0° and 12.0° at the beginning of each trial. To induce a trapezoid-like perspective in the illusion, two converging lines were shown with 4° separating them at the apex and 18° at the base. The total height of the imaginary trapezoid was 14.4° and the reference and adjustable segments were 11.3° away from each other. The whole illusion was centrally displayed.

The second variant of the PZ illusion (PZh), also called the corridor illusion, was inspired by previously referenced work (Schwarzkopf et al., 2011). Participants were instructed to adjust the size of the lower-left disk so that it appeared to be the same size as the upper-right disk or vice versa (Figure 1M). A tunnel image (640 × 480 pixels) was displayed in the center of the screen on a black background. The reference disk was 1° in diameter and the size of the adjustable disk was set in the range of 0° to 2° in diameter at the beginning of each trial. The adjustable disk was fixed at its lowest point, as if it was anchored to the image background.

##### TT illusion

Participants had to adjust the orientation of the right disk to match the orientation of the left inside disk or vice versa (Figure 1N). The reference and adjustable disks were 6.0° in diameter and made of a 0.5 cycles/° full contrast grating texture. The reference disk was titled 33° clockwise compared with the vertical and displayed with a random orientation at the beginning of each trial. The outside left disk was 20.0° in diameter and titled 36.0° counterclockwise compared with the vertical (0.5 cycles/° full contrast grating texture). The left and right disks were displayed 6.98° to the left and right compared with the middle of the screen. This illusion corresponded conceptually with the orientation contextual illusion used in previously cited work (Song et al., 2013), although the dimensions of the stimulus were different.

##### Zöllner (ZN) illusion

Participants were instructed to adjust the orientation of one main stream so that it appeared to be parallel to the other main stream (Figure 1O). The main streams were 16.60° long, 9.94° apart from each other, and tilted by 30.0° compared with the vertical. At the beginning of each trial, the adjustable main stream was randomly tilted between 0° and 90° compared with the vertical. Seven segments, 4.15° long, were intersecting each main stream. They were tilted 25° with respect to the main streams and their position relative to the main stream was randomly shifted between ±0.83°.

#### pRF mapping stimulus

The pRF mapping procedure was based on the one suggested by Alvarez, De Haas, Clark, Rees, and Schwarzkopf (2015) to yield a comparatively high pRF model fit among the other commonly used stimulus configurations. The stimulus consisted of a simultaneous rotating wedge and expanding and contracting ring (Supplementary Figure S1). The dimensions of the wedge and ring as well as the timing parameters were very similar to those described by van Dijk, de Haas, Moutsiana, and Schwarzkopf (2016). Each session ended with a 45-second period of fixation, with a total of 235 volumes acquired.

The wedge systematically stimulated different polar angles segments of the visual field, rotating around the fixation in 60 discrete steps of 6°. The wedge completed three cycles per scanning session. Independently, the ring systematically stimulated different eccentricity bands by expanding and contracting consecutively. The ring completed five cycles per session. The steps of the wedge and ring were synchronized with the start of each volume of the fMRI acquisition, that is, they lasted 1 second each. The wedge and ring aperture revealed a ripple background texture (as previously described; Alvarez et al., 2015; van Dijk et al., 2016), which changed every 500 ms. The ripple background consisted of black-and-white patterns, with a contrast of 100%. Outside of the aperture, the background of the display was set to a medium gray.

We included six sessions. In odd scanning sessions, the wedge rotated clockwise, and the ring had an expanding starting direction. In even sessions, this was reversed, with a counterclockwise ring rotation and contracting wedge starting direction.

The wedge stimulus was presented from 0.07° from fixation to an eccentricity of 12.00°, with an angular size of 12.00° at all eccentricities. As in previously referenced work (van Dijk et al., 2016), the diameter of the outer boundary of the ring was determined as a function of stimulus steps *x*:

$$\begin{array}{c} f\left(x\right)=17e^{\left(-4+\frac{1}{9}x\right)} \end{array}$$

The stimulus steps *x* increased or decreased with each acquired volume in steps of 0.9 from 1.5 to 33.0, yielding 36 discrete steps. The minimum eccentricity of the inner boundary of the ring stimulus was 0.25°; otherwise, the radius of the inner boundary was equal to 75% of the outer boundary.

Participants were instructed to always look at the fixation point (0.13° in diameter). To aid fixation, participants performed a simple fixation task. The fixation point changed color (red, green, white, black in pseudo-random order) with a stimulus onset asynchrony of 3 ± 1 seconds. Participants were asked to push a button within 1.5 seconds after each color change. The task was designed to keep the participant's gaze at the fixation point. Additionally, low-contrast crosshairs were presented to aid fixation. The crosshairs consisted of 12 radial lines from the fixation dot to the edge of the screen and 8 concentric circles, centered on fixation and increasing in radius in steps of 1.5°, starting from 2.0°.

### MRI data acquisition

Participants completed an MRI session directly following the behavioral session. MRI data were acquired using a 3 T whole-body MRI system (Magnetom Prisma, Siemens Medical Systems, Erlangen, Germany), using a 64-channel radiofrequency receive head coil and body coil for transmission. A high-resolution anatomical image (T1-weighted magnetization-prepared rapid gradient echo, 1.0-mm isotropic resolution) was acquired first and used to place the bounding boxes for the remaining sequences. Participants completed a practice run of the fMRI tasks during this acquisition. Functional T2*-weighted two-dimensional echoplanar images were acquired using a third-party multiband sequence (Moeller et al., 2010; Setsompop et al., 2012), with the following parameters: 235 volumes, 36 slices, 2.3-mm isotropic resolution, a repetition time of 1,000 ms, an echo time of 32.40 ms, a flip angle of 60°, a base resolution of 84 × 84, a 192-mm field of view, and a multiband factor of 4. A whole-brain echo planar imaging (EPI) volume was also acquired and used in an intermediate step in the spatial registration of the partial functional image with the anatomical image. Both the partial and whole-brain volumes consisted of axial slices that were tilted so that they were parallel to the calcarine sulcus for each participant. The central slice overlapped with the calcarine sulcus. A B0 field map was also acquired to allow for the correction of geometric distortions due to B0 field inhomogeneity in the EPI data (Hutton et al., 2002). The total acquisition time for all MRI sequences was approximately 70 minutes.

### MRI data analysis

#### Pre-processing

Cortical reconstruction was performed on the T1-weighted image using FreeSurfer's recon-all function (FreeSurfer software package [Dale, Fischl, & Sereno, 1999] version 6.0, http://surfer.nmr.mgh.harvard.edu/).

fMRI data pre-processing was done using the statistical parametric mapping (SPM) software package (SPM12, Wellcome Trust Centre for Neuroimaging, London, UK; http://www.fil.ion.ucl.ac.uk) in MATLAB. The data consisted of six experimental sessions of pRF mapping. The functional images were spatially realigned to the mean of the whole time-series using rigid-body transformations to correct for head motion. The B0 field map image was used to correct EPI geometric distortions. We then performed slice timing correction and intensity bias correction. The mean fMRI volume was co-registered first to the whole-brain fMRI in an intermediary step and then to the anatomical (T1-weighted) image using mutual information.

#### pRF mapping analysis

pRF mapping was done using the SamSrf 9.4 toolbox for pRF modelling (Schwarzkopf, 2023). The procedure included projecting the pre-processed retinotopic mapping fMRI data onto the cortical surface and then fitting a standard Gaussian two-dimensional pRF model to the data. The cortical surfaces were the outputs of FreeSurfer's *recon-all*.

pRF mapping was done by averaging the three odd (clockwise wedge, expanding ring) and three even (counterclockwise wedge, contracting ring) runs separately and then concatenating the two averaged runs. An occipital cortex region of interest (ROI) was used to restrict the number of surface vertices considered by the mapping algorithm. We used the outputs of the pRF mapping procedure—maps of eccentricity, polar angle, tuning width (standard deviation of the Gaussian) as a measure of pRF size, and variance explained (*R^2^*)—for further analysis.

We delineated visual area ROIs (V1 dorsal and ventral [V1d and V1v, respectively] V2d/v, V3d/v, and V4) based on reversals in the polar angle map and restricted our ROIs to realistic eccentricities (i.e., those that were mapped with the stimulus: 0° to 12°) using the eccentricity maps. For delineation purposes, we used a goodness-of-fit (*R*^2^) threshold of 0.1. To exclude noisy estimates near the stimulus edge, we restricted our further analyses to a maximum eccentricity of 8°. This was also the maximum eccentricity used in the studies we sought to replicate (Schwarzkopf et al., 2011; Schwarzkopf & Rees, 2013). Notably, to avoid any potential experimenter bias in the manual delineations, we used a blinded approach—M.A.J. and A.O. delineated the ROIs and were neither involved in the analysis of behavioral data (done by A.F.C.) nor did they at any point view individual participant's illusion data.

We used the following procedure (outlined in previous work; Hofstetter, Cai, Harvey, & Dumoulin, 2021) to convert the goodness of fit (*R^2^*) of the pRF models to the probability of observing the model fits by chance: we did pRF mapping as outlined above but fitting the signals of about 84k cortical vertices outside of the occipital cortex mask that was used for the analysis proper but on the same scans. The rationale was that the pRF mapping fit outside the occipital cortex should be quite poor and would therefore constitute a null distribution of goodness-of-fit values. Further analyses only included those cortical vertices for which the probability of observing the given goodness-of-fit value by chance was less than 0.05. This corresponded with an *R^2^* threshold of 0.07. In the pRF size analyses, we further controlled for the model fitting-related data quality by weighting the contribution of cortical vertices by the corresponding *R^2^* value.

### Data analysis

#### Illusion magnitudes

Analyses of illusion data were performed in R (R Development Core Team, The R Foundation for Statistical Computing, Vienna, Austria). As a measure of intrarater reliability, we computed two-way mixed effects models (intraclass correlations of type [3,1] or ICC_3,1_) between the two adjustments of each condition (Koo & Li, 2016; Shrout & Fleiss, 1979). More than a simple correlation, ICCs also reflect the agreement between measurements. As suggested by Gignac and Szodorai (2016), we considered correlation coefficients of 0.1, 0.2, and 0.3 as small, medium, and large, respectively. Note, however, that Cohen (1988) suggested considering 0.1, 0.3, and 0.5 as the cut-off values for small, medium, and large effect sizes.

The illusion magnitude (averaged across two trials) was expressed as a difference compared with the reference, except for the HC and EX conditions, where the extracted value was the area of the adjusted ellipse. For each participant and each condition, we combined both conditions of each illusion, that is, we added the additive inverse value of the condition that is usually underadjusted (this was usually a negative number, so taking the additive inverse usually made the value positive) to the value of the overadjusted condition. Correlations were then computed between each pair of illusions to inspect individual differences in the perception of visual illusions.

#### Visual surface area

In a first analysis, we calculated the surface area of visual areas V1, V2, V3, and V4 within the eccentricities bounded by the foveal representation and 8°, summing the areas of all the cortical vertices within this eccentricity range. We averaged the surface areas of the two hemispheres. We then estimated the correlations between illusion magnitude and the ROI surface area for each illusion–ROI pair. We detected outliers based on the absolute modified *z*-score, which is more robust than the common *z*-score because it is based on the median and median absolute deviation instead of the mean and standard deviation. Any data point that had an absolute modified *z*-score of greater than 3.5 in terms of either illusion magnitude or ROI surface area was removed, as suggested by Iglewicz and Hoaglin (1993).

We used the open-source JASP software (Jeffreys's Amazing Statistics Program; https://jasp-stats.org; Love et al., 2019) for Bayesian and frequentist correlation analyses of illusion magnitudes and ROI surface areas. We considered correlation coefficients of 0.1, 0.3, and 0.5 as small, medium, and large effect sizes, respectively (Cohen, 1988). We considered a BF_10_ between 3 and 10 to correspond with substantial evidence in favor of the alternative hypothesis (Jeffreys, 1961) (in this case, substantial correlation between the two variables).

#### pRF size

The previously mentioned studies that found correlations between size illusion magnitude and V1 surface area argued that their findings could be explained by the relation between subjective size perception and the spatial spread of neural activity (Moutsiana et al., 2016; Schwarzkopf et al., 2011; Schwarzkopf & Rees, 2013). Although our analysis used a functionally defined surface area as a measure of central cortical magnification across the visual field, we wanted to investigate the role of the local spatial spread of neural activity.

Thus, in our second analysis, we estimated pRF sizes at illusion-relevant eccentricities. For each participant, each ROI and the two hemispheres separately, we fit a linear regression model to pRF size as a function of eccentricity, weighted by the pRF model's goodness of fit (*R^2^*) at each given vertex. Fitting was done for eccentricities between 1° and 8° to remove edge-of-stimulus effects (Supplementary Figure S2 provides an example). We computed the slope and intercept of the least squares line and used them to estimate the pRF size at the illusion-relevant eccentricity.

We then computed the correlations between these pRF sizes and the corresponding illusion magnitudes. This analysis can only be done for illusions in which the targets are at a fixed distance from one another. Moreover, the distance between the targets can only be reasonably determined for circular targets. Therefore, here we focused on the DB and EB2 illusions. In both cases, we assumed that participants used one of two strategies: 1) focusing their gaze at the midpoint location between the two targets, or 2) focusing their gaze directly on one or the other of the targets.

In the DB illusion, the distance between the centers of the two targets was 3.92°, with the targets positioned diagonally from each other—one in the upper left and the other in the lower right. Thus, we estimated pRF sizes from the following eccentricities and ROIs: right ventral (corresponding with the top left target) and left dorsal (corresponding with the bottom right target) ROIs, at eccentricities of 3.92° and 1.96° (=3.92°/2; i.e., the midpoint between the two target locations). Similarly, for the EB2 illusion, we considered the right and left hemisphere ROIs (because targets were positioned horizontally from each other), at eccentricities of 9.3° (the distance between the targets) and 4.65° (the midpoint between the targets).

We calculated the Bayesian and frequentist correlations between the DB and EB2 illusion magnitudes and pRF sizes at the aforementioned eccentricities for each ROI. We removed any data point that had a modified *z*-score of greater than 3.5 in terms of either illusion magnitude or pRF size (Iglewicz & Hoaglin, 1993). As discussed, we considered correlation coefficients of 0.1, 0.3, and 0.5 as small, medium, and large effect sizes, respectively, and a BF_10_ between 3 and 10 as substantial evidence in favor of the alternative hypothesis.

#### Slope and intercept of pRF size as a function of eccentricity

Our pRF analysis was limited to just two illusions due to the difficulty of identifying relevant eccentricities for most illusions in a free-viewing condition and with noncircular targets. To circumvent this issue and characterize the relationship between pRF size and illusion magnitude for all tested illusions, we conducted a final analysis, in which we estimated the slope and intercept of pRF size as a function of eccentricity. An association between illusion magnitude and the slope would indicate that interindividual susceptibility to the given illusion is predicated on the rate at which pRF size increases with eccentricity. Meanwhile, an association between illusion magnitude and the intercept would imply that the parafoveal pRF size is the determining factor for illusion susceptibility.

As in the pRF size analysis, we fit a linear regression model to pRF size as a function of eccentricity (for eccentricities between 1° and 8°), weighted by the pRF model's goodness of fit (*R^2^*). We then averaged the resulting slopes and intercepts from the two hemispheres. For some participants, the slope was estimated to be negative due to noise in the data (Supplementary Figure S3). If this was the case in one hemisphere, we only considered the slope from the other hemisphere. If it was the case in both hemispheres, we omitted the data point from further analysis.

For each illusion and each ROI, we conducted Bayesian linear regression analyses, with illusion magnitudes as the dependent variable and slope and/or intercept as the covariate(s). We tested four models: 1) a model that included the slope, 2) a model that included the intercept, 3) a model that included both the slope and the intercept, and 4) a null model that did not include either the slop or the intercept. We estimated the Bayes factors in favor of each of the three alternative models over the null model. As discussed, we considered a BF_10_ between 3 and 10 to correspond with substantial evidence in favor of the alternative model.

## Results

### Illusion magnitudes

We tested 30 normally sighted participants on 13 visual illusions: BS, CS, DB, two variants of the EB (EB1 and EB2), EX, HC, ML, PD, two variants of the PZ (PZ and PZh), TT, and ZN illusions (Figure 1). The Methods provide details on each illusion.

To test illusion magnitudes, we used an adjustment procedure, in which the participants manually adjusted a target element with a computer mouse. Each illusion was tested with two reference-dependent conditions. For example, in the BS illusion, participants were asked to adjust the length of the vertical segment to match the length of the horizontal segment or vice versa. Participants right clicked the mouse to validate a trial. Each condition was tested twice, with all illusions presented in random order. Unless otherwise specified, illusions were tested under free viewing conditions, that is, without a fixation point. Illusions were tested in a behavioral testing room, outside of the MRI scanner.

As a measure of test–retest reliability, ICCs were computed between the two adjustments of each condition (see Methods for details). All ICC coefficients were strong and significant (Supplementary Table S1), except for the CS illusion (CS left and CS right), which showed small effect sizes.

We calculated illusion magnitudes as the difference compared with the reference, except for the HC and EX illusions, where the area of the adjusted ellipse was considered as a measure of the illusion magnitude. Supplementary Figure S4 and Supplementary Table S2 show the illusion magnitudes for both conditions of each illusion. All illusions, except the EX and HC illusions, were overadjusted in one reference-dependent condition and underadjusted in the other. For example, the horizontal segment of the BS illusion was overadjusted when compared with the vertical segment, and the vertical segment was underadjusted when compared with the horizontal segment.

We combined the two magnitudes of each illusion, that is, the additive inverse of the illusion magnitude of the condition that is usually underadjusted was added to the illusion magnitude of the condition that is usually overadjusted. We then calculated between-illusion correlations (Table 1). In general, most correlations were weak, except for the correlations between the magnitudes of two variants of the same illusion, for example, between the EB1 and EB2 illusions or between the PZ and PZh illusions. We also observed a strong correlation between the EX and HC illusion magnitudes, which suggests that a similar mechanism underlies the two illusions, as previously reported (Bertamini et al., 2019).

**Table 1.** Between-illusion correlation coefficients (Pearson's *r*). *Notes*: Upper triangle: the color scale from blue to red reflects effect sizes from *r* = −1 to *r* = 1. Lower triangle: bold and bold italic fonts indicate significant results without (*α* = 0.05) and with (*α* = 0.05/78) Bonferroni correction, respectively.

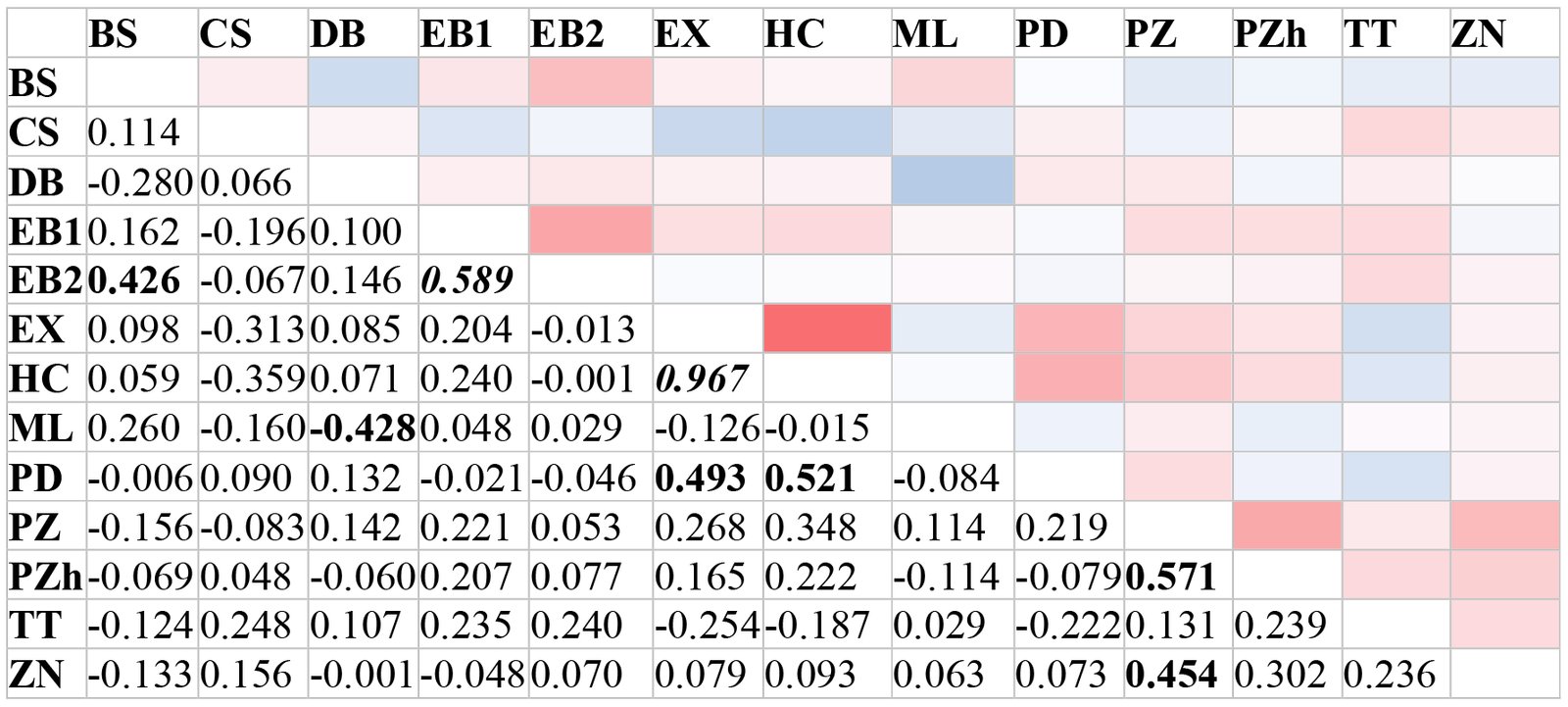

We additionally examined whether the lack of strong correlations could be explained by measurement reliability, computing noise ceilings from the ICCs of each illusion. As shown in Supplementary Figure S5, the vast majority of low to medium correlations (|*r*| < 0.5) were well below their respective noise ceilings, with only three illusion pairs lying within 10% of their ceilings. Notably, these exceptions were driven by the relatively low reliability of a single illusion (CS illusion). On average, observed correlations were approximately 31% lower than their respective ceilings, suggesting that measurement noise may contribute but is unlikely to be the primary limiting factor in detecting correlations between the illusions.

### Cortical idiosyncrasies and illusion magnitude

All participants completed an MRI session immediately after the behavioral session. The MRI session included six runs of pRF mapping and a high-resolution anatomical scan. We used a wedge-and-ring stimulus for pRF mapping, which has been shown to result in a higher model fit and to necessitate shorter acquisition times in comparison with other stimulus configurations (Alvarez et al., 2015). We manually delineated visual areas V1, V2, V3, and V4 on the resulting polar angle maps. We then estimated measures of cortical idiosyncrasy (surface area and pRF size) for each of the visual areas and correlated these measures with the illusion magnitudes obtained for each participant. See Methods for details.

#### Visual surface area

Schwarzkopf et al. reported negative correlations between the retinotopically defined surface area of the primary visual cortex V1 and the EB (Schwarzkopf et al., 2011; Schwarzkopf & Rees, 2013), PZh (Schwarzkopf et al., 2011), and TT (Song et al., 2013) illusion magnitudes and between V1 pRF size and DB illusion magnitude (Moutsiana et al., 2016). To replicate their findings, we first calculated the surface area of visual areas V1, V2, V3, and V4 within the eccentricities bounded by the foveal representation and an eccentricity of 8°, combining the surface areas of the left and right hemispheres. As expected, the surface areas of visual areas V1 to V4 decreased up the visual hierarchy (mean for V1, 2.577e+3 ± 648.673 mm^2^; for V2, 2.205e+3 ± 530.511 mm^2^; for V3, 1.716e+3 ± 368.319 mm^2^; and for V4, 0.820e+3 ± 213.605 mm^2^), and the values were consistent with previous reports. Also as expected, surface areas of adjacent visual areas were highly correlated (V1–V2: *r* = 0.803, *p* = 9.177e-8; V2–V3: *r* = 0.572, *p* = 9.713e-4; V3–V4: *r* = 0.515, *p* = 0.004; and V1–V3: *r* = 0.501, *p* = 0.005). We correlated the surface areas with illusion magnitudes from the 13 tested illusions. Here, we group the illusions as follows: 1) illusions previously studied by Schwarzkopf and colleagues (DB, EB2, PZh, and TT illusions), 2) other size illusions (BS, EB1, ML, and PZ illusions), and 3) uniform texture, perceived orientation, and contrast illusions (CS, EX, HC, PD, and ZN illusions). The illusion magnitude–surface area correlations are plotted in separate figures for each of these three groups.

Illusion magnitude–surface area correlations for illusions previously studied by Schwarzkopf et al. are shown in Figure 2. Bayes factors in favor of the alternative hypothesis (BF_10_) for each correlation are reported in Table 2, along with Pearson's *r*. We interpreted the strength of evidence in favor of the null or alternative hypothesis based on the following BF_10_ cutoffs (Jeffreys, 1961; Lee & Wagenmakers, 2013): substantial evidence for the null hypothesis, BF_10_ ∈ [0.1,0.333); anecdotal evidence for the null hypothesis, BF_10_ ∈ [0.333,1); anecdotal evidence for the alternative hypothesis, BF_10_ ∈ [1,3); substantial evidence for the alternative hypothesis, BF_10_ ∈ [3,10); and extreme evidence for the alternative hypothesis (BF_10_ > 100). In general, our results failed to replicate those reported by Schwarzkopf et al. We did not observe a significant negative correlation between the DB illusion magnitude and V1 surface area, with the Bayes factor in fact indicating substantial evidence in favor of the null hypothesis (BF_10_ = 0.313). We observed medium effect sizes for the negative correlations in the other ROIs, with a significant correlation in the case of V2 (*p* < 0.05, uncorrected), but the Bayes factors did not indicate substantial evidence in favor of the alternative hypothesis in any of the ROIs (V2, *r* = −0.373, *p* = 0.043, BF_10_ = 1.616; V3, *r* = −0.336, *p* = 0.074, BF_10_ = 1.052; and V4, *r* = −0.320, *p* = 0.090, BF_10_ = 0.905). For the EB2 illusion, we did not observe any negative correlations between illusion magnitude and the visual surface area. In fact, we found (weak) *positive* correlations in visual areas V2 to V4 and substantial evidence in favor of the null hypothesis across all ROIs (V1, *r* = 0.168, *p* = 0.384, BF_10_ = 0.238; V2, *r* = 0.113, *p* = 0.558, BF_10_ = 0.242; V3, *r* = 0.019, *p* = 0.922, BF_10_ = 0.240; and V4, *r* = 0.131, *p* = 0.506, BF_10_ = 0.245). We also did not observe any significant correlations for the PZh illusion. Indeed, the Bayes factors across all ROIs indicated substantial evidence in favor of the null hypothesis (V1, *r* = 0.005, *p* = 0.979, BF_10_ = 0.235; V2, *r* = 0.112, *p* = 0.569, BF_10_ = 0.274; V3, *r* = −0.148, *p* = 0.461, BF_10_ = 0.309; and V4, *r* = 0.128, *p* = 0.526, BF_10_ = 0.289). Finally, for the TT illusion, we did not observe any significant correlations, with factors indicating substantial evidence in favor of the null hypothesis in V3 (BF_10_ = 0.265) and V4 (BF_10_ = 0.237).

**Figure 2.**
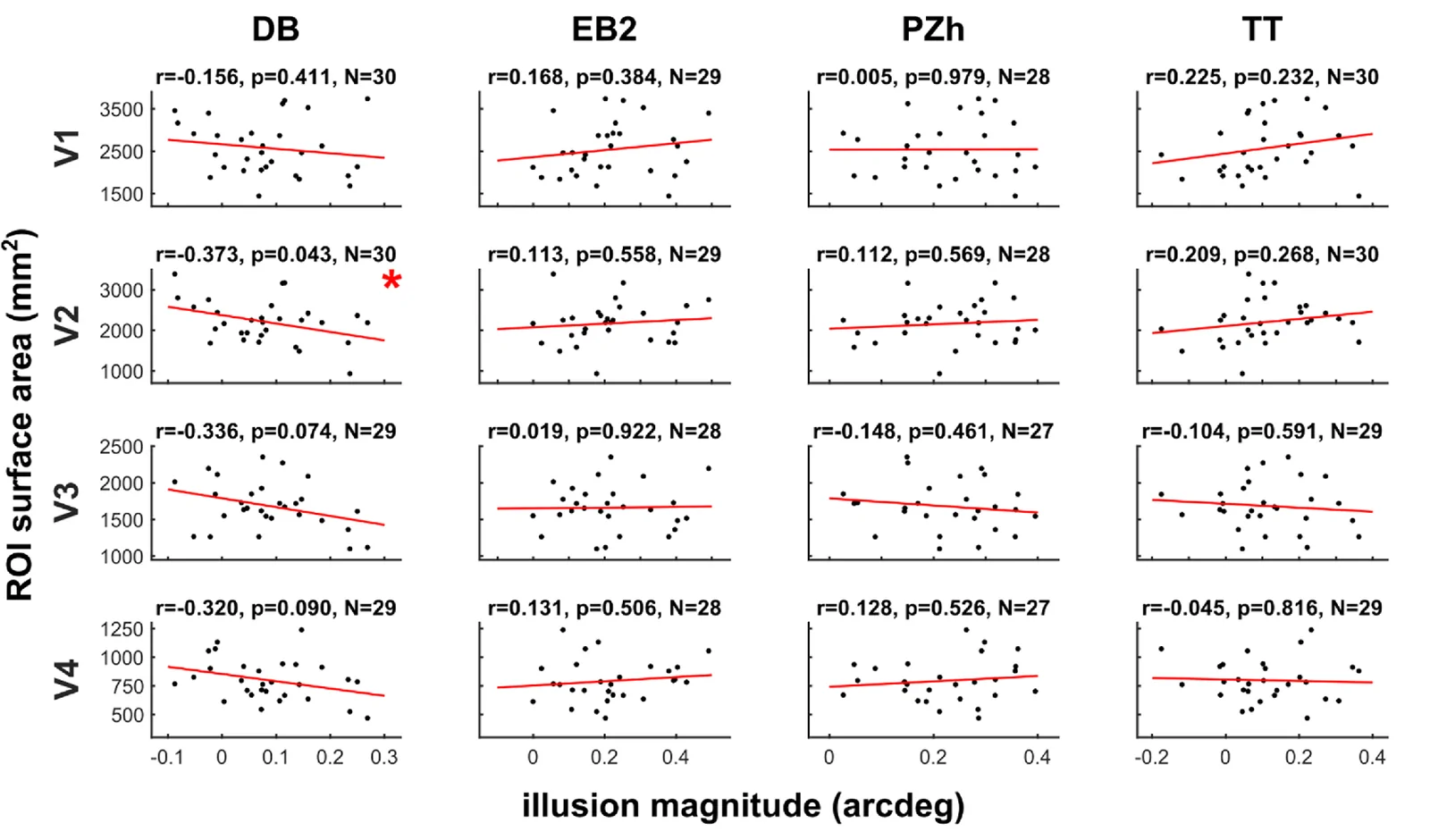
Correlations between the ROI surface area and the visual illusion magnitudes for illusions previously studied by Schwarzkopf et al.: the DB illusion as in Moutsiana et al. (2016); the EB2 illusion as in Schwarzkopf & Rees, (2013); the PZh illusion as in Schwarzkopf et al. (2011); and the TT illusion as in Song et al. (2013). An asterisk marks the one significant correlation (*p* < 0.05, uncorrected). Outliers (absolute modified *z*-score > 3.5) were removed.

**Table 2.** Correlations between the ROI surface areas (mm^2^) and illusion magnitudes. *Notes*: Significant correlations (*p* < 0.05, uncorrected) are marked in bold with an asterisk. Only one of these correlations constitutes at least substantial evidence in favor of the alternative model. In fact, this correlation—between the V3 surface area and the BS illusion magnitude—constitutes extreme evidence in favor of the alternative model (BF_10_ > 100) and is marked in bold with three asterisks. Instances of substantial evidence in favor of the null hypothesis (BF_10_ ∈ [0.1,0.333) are marked in bold with a dagger (†). Outliers (absolute modified *z*-score > 3.5) were removed.

Visual area	Illusion	No.	Pearson's *r*	BF₁₀
V1	BS	30	0.317	0.912
	Contrast	30	0.189	0.366
	DB	30	−0.156	**0.313^†^**
	EB1	30	0.170	0.334
	EB2	28	0.032	**0.238^†^**
	EX	29	−0.172	0.337
	HC	30	−0.074	**0.244^†^**
	ML	29	0.168	**0.331^†^**
	PD	29	−0.251	0.526
	PZ	30	−0.101	**0.259^†^**
	PZh	28	0.005	**0.235^†^**
	TT	30	0.225	0.449
	ZN	30	−0.132	**0.286^†^**
V2	BS	30	0.360	1.397
	CS	30	0.122	**0.277^†^**
	DB	30	−0.373*	1.616
	EB1	30	0.211	0.413
	EB2	28	0.051	**0.242^†^**
	EX	29	−0.164	**0.326^†^**
	HC	30	0.074	**0.244^†^**
	ML	29	0.113	**0.272^†^**
	PD	29	−0.343	1.127
	PZ	30	−0.138	**0.292^†^**
	PZh	28	0.112	**0.274^†^**
	TT	30	0.209	0.407
	ZN	30	−0.248	0.523
V3	BS	29	0.628*	130.580***
	CS	29	0.136	**0.292^†^**
	DB	29	−0.336	1.052
	EB1	29	0.185	0.358
	EB2	27	0.016	**0.240^†^**
	EX	28	−0.018	**0.236^†^**
	HC	29	0.105	**0.265^†^**
	ML	28	0.019	**0.236^†^**
	PD	28	−0.386*	1.656
	PZ	29	−0.200	0.386
	PZh	27	−0.148	**0.309^†^**
	TT	29	−0.104	**0.265^†^**
	ZN	29	−0.419*	2.633
V4	BS	29	0.272	0.611
	CS	29	0.010	**0.231^†^**
	DB	29	−0.320	0.905
	EB1	29	0.158	**0.318^†^**
	EB2	27	0.048	**0.245^†^**
	EX	28	−0.037	**0.239^†^**
	HC	29	−0.136	**0.292^†^**
	ML	28	0.131	**0.290^†^**
	PD	28	−0.201	0.387
	PZ	29	−0.198	0.383
	PZh	27	0.128	**0.289^†^**
	TT	29	−0.045	**0.237^†^**
	ZN	29	−0.048	**0.237^†^**

Supplementary Figure S6 shows the illusion magnitude–surface area correlations for the other size illusions (the BS, EB1, ML, and PZ illusions). Here, we did not observe any significant negative correlations. We observed weak negative correlations for the ML and PZ illusions across ROIs, but none of them were significant. Contrary to the observation of negative correlations between V1 surface areas and size illusion magnitudes by Schwarzkopf et al., we observed *positive* correlations for the BS illusion across visual areas, although the correlation was significant only in V3 (*r *= 0.628, *p* = 2.62e-4), with extreme evidence (BF > 100) in favor of the alternative hypothesis (BF_10_ = 130.580) (Jeffreys, 1961; Lee & Wagenmakers, 2013). Likewise, we observed weak positive correlations for the EB1 illusion across all ROIs, although none of these correlations were significant.

Illusion magnitude–surface area correlations for all other illusions (uniform texture, perceived orientation, and contrast illusions) are shown in Supplementary Figure S7. We observed two significant correlations between illusion magnitude and visual surface area, one for the PD illusion in V3 (*r* = −0.386, *p* = 0.043, BF_10_ = 1.656) and another for the ZN illusion in well (*r* = −0.419, *p* = 0.024, BF_10_ = 2.633); however, neither of these constituted substantial evidence in favor of the alternative hypothesis.

#### pRF size and illusion magnitude

Previous studies have investigated the role of macroscopic visual surface area in determining interindividual variability in perception under the assumption that it is a proxy for local variations in cortical magnification or, inversely, pRF size. The initial seminal studies linking illusion susceptibility with early visual surface areas (Schwarzkopf et al., 2011; Schwarzkopf & Rees, 2013) relied on phase-encoded methods rather than model-based (Dumoulin & Wandell, 2008) retinotopic mapping, which—although it allowed for a manual delineation of early visual areas based on reversals in the polar angle map—did not allow for the estimation of pRF size. Here, we used pRF modeling to directly investigate the role of pRF size in determining illusory size perception. Moreover, we sought to replicate the findings from the other previously mentioned study by Moutsiana et al. (2016), which used model-based pRF mapping and reported a negative correlation between pRF size and DB illusion magnitude. To this end, we extracted pRF sizes from eccentricities relevant to each given illusion and correlated them with illusion magnitudes.

This analysis could only be done for illusions in which the targets are at a fixed distance from one another. Moreover, the distance between the targets could be reasonably determined only for circular targets. Thus, we focused on the DB and EB2 illusions. In both cases, we assumed that participants used one of two strategies: 1) focusing their gaze at the midpoint location between the two targets, or 2) focusing their gaze directly on one or the other of the targets. We extracted pRF sizes from illusion-relevant eccentricities based on the linear fit of the pRF size–eccentricity relationship, in accordance with both strategies (see Methods for details). From this point forward, we refer to the eccentricities used in strategy 1 as the larger eccentricity and strategy 2 as the smaller eccentricity.

Correlations between illusion magnitudes and pRF sizes at illusion-relevant eccentricities are shown in Figure 3 for the DB illusion and in Figure 4 for the EB2 illusion. The Bayes factor in favor of the alternative hypothesis (BF_10_) for each correlation is reported in Table 3, along with Pearson's *r*. In general, we failed to observe an association between pRF size and illusion magnitude. For the DB illusion, we observed two medium effect size *positive* correlations in V3, one in the case of the smaller eccentricity (1.96°, *r* = 0.341, *p* = 0.066, BF_10_ = 1.145) and the other in the case of the larger eccentricity (3.92°, *r* = 0.360, *p* = 0.051, BF_10_ = 1.404). Neither of the two was significant and constituted only anecdotal evidence in favor of the alternative hypothesis. Similarly, for the EB2 illusion, we observed two correlations of medium effect size in V3 (*r* = 0.319, *p* = 0.099, BF_10_ = 0.861) and V4 (*r* = 0.444, *p* = 0.016, BF_10_ = 3.697), both for the smaller eccentricity (4.65°). Only the latter correlation was significant, with the Bayes factor indicating substantial evidence in favor of the alternative hypothesis.

**Figure 3.**
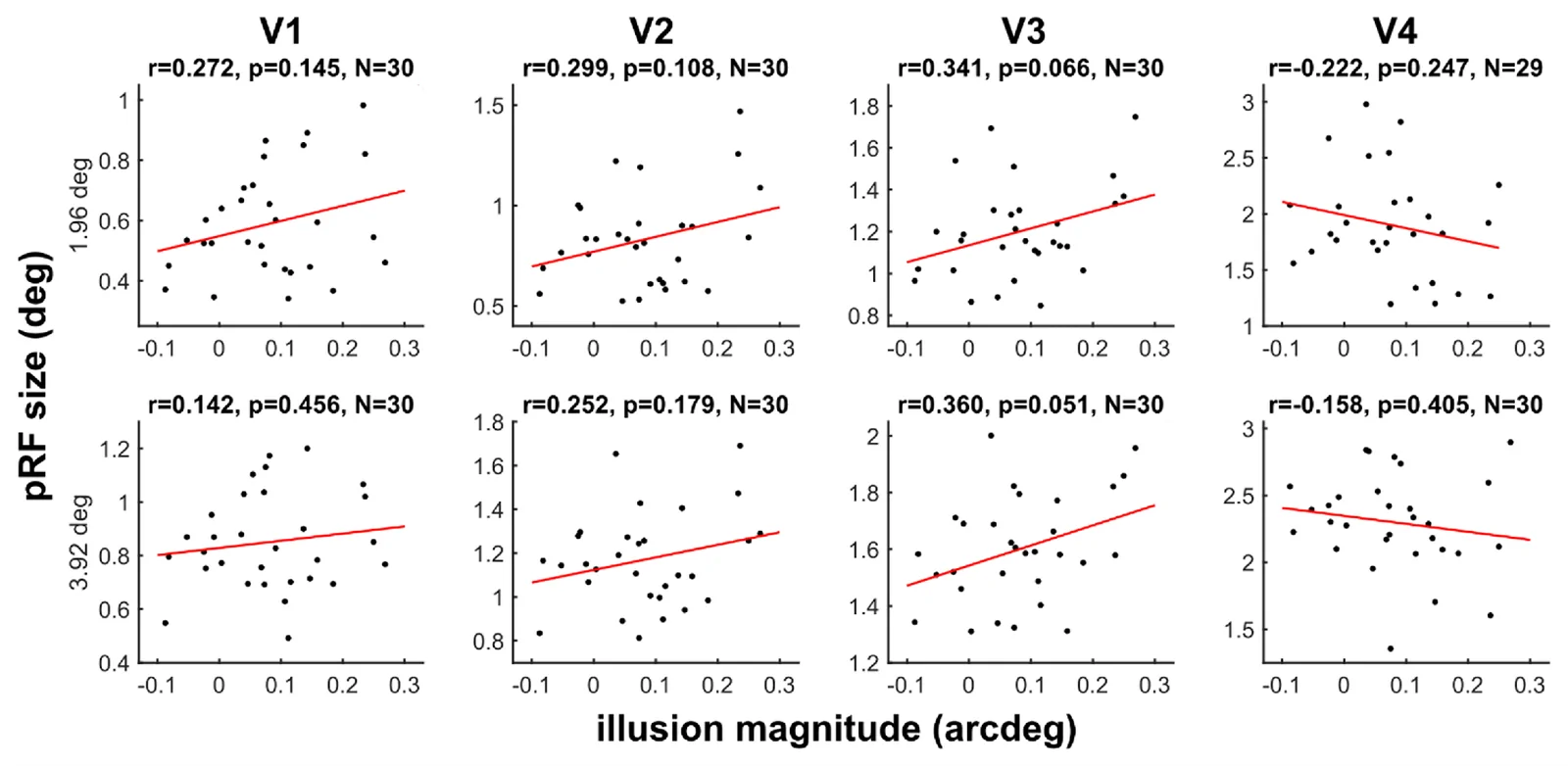
Correlations between pRF size at the illusion-relevant eccentricities and the visual illusion magnitudes for the DB illusion. None of the correlations are significant (*p* < 0.05, uncorrected).

**Figure 4.**
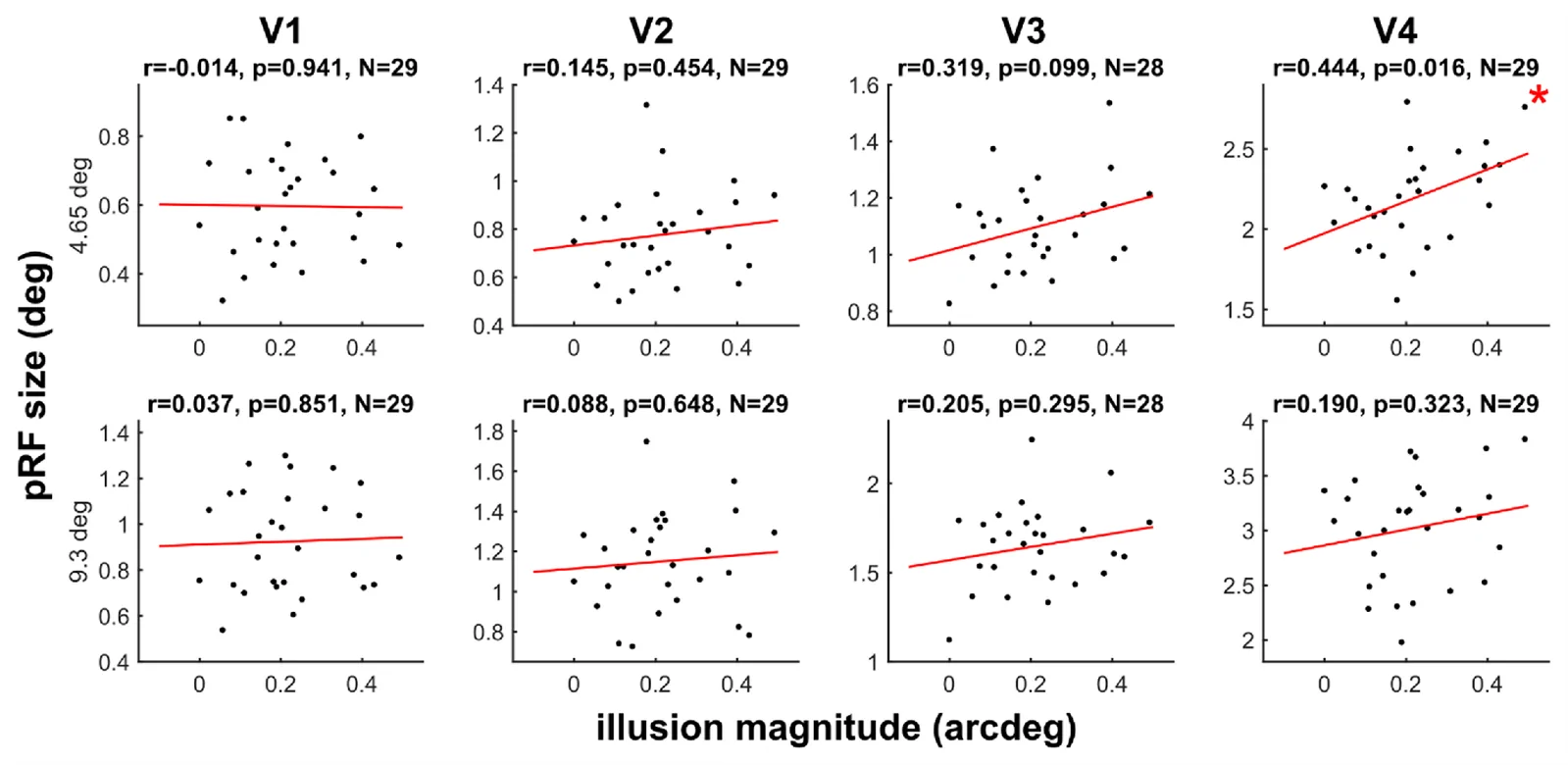
Correlations between pRF size at the illusion-relevant eccentricities and visual illusion magnitudes for the EB2 illusion. An asterisk marks the one significant correlation (*p *< 0.05, uncorrected for multiple comparisons).

**Table 3.** Correlations between pRF size (in degrees) at relevant eccentricities and illusion magnitudes with the outliers removed. *Notes*: The one significant correlation (*p* < 0.05, uncorrected) is marked in bold with an asterisk. Only this single correlation constitutes substantial evidence in favor of the alternative model (BF_10_ ∈ [3,10]). Numerous correlations constitute substantial evidence in favor of the null hypothesis (BF_10_ ∈ [0.1,0.333])—these are marked in bold with a dagger (†).

Visual area	Illusion	Eccentricity (°)	No.	Pearson's *r*	BF₁₀
V1	DB	1.96	30	0.272	0.625
		3.92	30	0.142	**0.296^†^**
	EB2	4.65	29	−0.014	**0.231^†^**
		9.3	29	0.037	**0.235^†^**
V2	DB	1.96	30	0.299	0.779
		3.92	30	0.252	0.538
	EB2	4.65	29	0.145	**0.302^†^**
		9.3	29	0.088	**0.255^†^**
V3	DB	1.96	30	0.341	1.145
		3.92	30	0.360	1.404
	EB2	4.65	28	0.319	0.861
		9.3	28	0.205	0.395
V4	DB	1.96	29	−0.222	0.438
		3.92	30	−0.158	**0.316^†^**
	EB2	4.65	29	0.444*	3.697*
		9.3	29	0.190	0.368

#### Slope and intercept of pRF size as a function of eccentricity

Our pRF analysis was limited to just two illusions due to the difficulty of identifying relevant eccentricities for most illusions. To circumvent this issue and characterize the relationship between pRF size and illusion magnitude for all the tested illusions, we estimated the slope and the intercept of pRF size as a function of eccentricity. An association between the slope and illusion magnitude would indicate that interindividual susceptibility to the given illusion is predicated on the rate at which pRF size increases with eccentricity. Meanwhile, an association between the intercept and illusion magnitude would imply that the parafoveal pRF size is the determining factor for illusion susceptibility.

For each participant, each ROI, and the two hemispheres separately, we fit a linear regression model to the pRF size as a function of eccentricity, weighted by the pRF model's goodness of fit (*R*^2^) at each given eccentricity. We calculated the slope and the intercept of the regression line and averaged the slopes and intercepts from the two hemispheres (for further details, see the Methods).

For each illusion and each ROI, we conducted Bayesian linear regression analyses, with illusion magnitudes as the dependent variable and slope and/or intercept as the covariate(s). We tested four models: 1) a model that included the slope, 2) a model that included the intercept, 3) a model that included both the slope and the intercept, and 4) a null model that included neither the slope nor the intercept. Bayes factors in favor of each of the three alternative Bayesian linear regression models—slope, intercept, and slope plus intercept—are reported in Supplementary Table S3. We did not find substantial evidence for any of the tested models with respect to the null model for any of the tested illusions. For most of the other slope and intercept models, we found anecdotal evidence for the null hypothesis. Meanwhile, for the model containing both the slope and the intercept terms, we found substantial evidence in favor of the null model for most illusions and ROIs (BF_10_ ∈ [0.1,0.333]).

## Discussion

Susceptibility to visual illusions is highly variable across individuals. Here, we investigated whether individual differences in the perception of visual illusions could be linked to individual differences in early visual surface area and pRF size. Our findings speak against the existence of common factors, both at the behavioral and neural levels. Across a battery of 13 illusions, we found little to no correlation between illusion magnitudes, reinforcing the idea that illusion susceptibility is largely independent across different types of illusions. Furthermore, we did not replicate previous findings of correlations between illusion magnitudes and early visual surface areas or pRF size (Moutsiana et al., 2016; Schwarzkopf et al., 2011; Schwarzkopf & Rees, 2013; Song et al., 2013). We found almost no significant correlations. These findings challenge previous reports of variability in visual illusion perception being driven by neural common factors.

Intuitively, one might expect that someone with high susceptibility to the EB illusion has high susceptibility to the PZ illusion as well. Taxonomizing illusions as spatial, temporal, and contrast illusions reflects this thinking. For instance, Coren, Girgus, Erlichman, and Hakstian (1976) investigated the interrelationships between the susceptibility to 45 illusion configurations using factor analysis, identifying five primary factors. Yet nearly one-half of the tested illusions were variants of the ML illusion, with two of the five factors dominated by ML variants. In general, recent research clearly shows that, although different *variants* of the same illusion may form robust common factors, strong correlations between different illusions are rare (Cretenoud et al., 2019; Cretenoud, Francis, et al., 2020; Cretenoud, Grzeczkowski, et al., 2020; Grzeczkowski et al., 2017; Grzeczkowski et al., 2018; Mazuz et al., 2023; Takahashi et al., 2023); however, see Makowski, Te, Kirk, Liang and Chen (2023) and Yu, Fischer, and Schwarzkopf (2025). Schwarzkopf et al. also reported that the illusion magnitudes in the EB and PZ illusions did not correlate significantly (Schwarzkopf et al., 2011). The low correlations cannot be explained by low test–retest reliability, lack of power, or a limited range in the illusion magnitudes (Cretenoud et al., 2019; Grzeczkowski et al., 2017; Grzeczkowski et al., 2018). Factor analyses confirm these results and suggest that each illusion makes up its own factor (Cretenoud et al., 2019; Cretenoud, Barakat, et al., 2021; Cretenoud, Francis, et al., 2020; Cretenoud, Grzeczkowski, et al., 2020). It must be noted that, even though interillusion correlations are weak, the correlations between illusion susceptibility and neural measures could be sound, because correlations are only transitive when they are sufficiently close to 1 (Langford, Schwertman, & Owens, 2001).

For this reason, we sought to re-evaluate previous findings, conducting a systematic study with a variety of illusions. We did not replicate the previous findings of negative correlations between early visual surface areas and susceptibility to different size illusions (DB, EB2, TT, and PZh illusions). Almost none of the correlations were significant. Although we observed negative correlations for some selected illusions and brain regions (small-to-medium effect sizes), the rest of the correlations were mostly *positive* (small-to-medium effect sizes). The finding of positive correlations is surprising, because it suggests that larger visual cortical surface areas give rise to stronger illusion susceptibility, whereas Schwarzkopf et al. suggested that the mediating factor between illusion magnitude and cortical size is the spatial spread of neuronal connections between the target and inducer. In addition to the surface area analysis, we tested whether illusion susceptibility can be explained by pRF size (as in previously referenced work; Moutsiana et al., 2016) for the DB and EB2 illusions. We failed to replicate the previous findings, observing no significant correlations. In a final analysis, we tried to explain illusion susceptibility with the parafoveal pRF size and the rate of change of pRF size from the fovea to the periphery, using the intercept and slope of the linear relation between pRF size and eccentricity. Once again, we did not find any significant results.

Our study goes well beyond the previously mentioned studies (Moutsiana et al., 2016; Schwarzkopf et al., 2011; Schwarzkopf & Rees, 2013; Song et al., 2013). First, we tested a much larger battery of 13 visual illusions including 4 other size illusions—the BS illusion, another variant of the EB illusion, the ML illusion, and the PZ illusion—and six other illusions of uniform texture, perceived orientation, and contrast. Again, we found mainly weak correlations. A second important difference was that two of the aforementioned studies (Schwarzkopf et al., 2011; Schwarzkopf & Rees, 2013) used phase-encoded methods to functionally define visual surface areas as a measure of central visual cortical magnification. Here, we used model-based pRF mapping (Dumoulin & Wandell, 2008), which, in addition to providing more robust eccentricity measures, also estimates pRF size, that is, the spatial spread of the neural activation in response to the visual stimulus. This strategy allowed us to correlate illusion magnitudes not only with V1 surface area, but also with pRF size, as in previously referenced work (Moutsiana et al., 2016). Notably, we estimated pRF sizes using an *R*^2^-weighted linear regression of pRF size on eccentricity. Although designed to improve the robustness of pRF size estimates, the approach is limited in V4 due to the region's less defined retinotopic organization and fMRI signal artifacts. Third, in our study we used free-viewing conditions, which meant that we did not control the participants’ subjective experience of the illusions due to individual gaze patterns. As a result, we had to make assumptions about the viewing strategies that participants used. To account for any systematic biases that can result from the assumptions of the two strategies, future studies should include a fixation point. Fourth, we used an adjustment task, whereas the aforementioned studies used psychometric curve fitting and two-alternative forced choice or multiple alternative perceptual search tasks (Moutsiana et al., 2016; Schwarzkopf et al., 2011; Schwarzkopf & Rees, 2013; Song et al., 2013). However, we have previously shown that individual differences are stable across different measurement methods (Cretenoud, Grzeczkowski, et al., 2021). This fact was corroborated by the findings of one of the aforementioned studies, in which the authors compared illusion magnitudes obtained via psychometric fitting with those from an adjustment procedure and found very high correlations between the two measures for the EB and PZh illusions (Schwarzkopf et al., 2011). The reliability and robustness of our illusion measurements is also evidenced by the high test–retest reliability across all tested illusions. However, recent work from Yu et al. (2025) suggests that the adjustment procedure is less reliable than the two-alternative forced choice method, which may have contributed to the discrepancy between our findings and those reported previously (Moutsiana et al., 2016; Schwarzkopf et al., 2011; Schwarzkopf & Rees, 2013; Song et al., 2013).

There are increasing calls for large cohort studies or individual-focused precision studies due to reproducibility and statistical power concerns (Gordon et al., 2017; Poldrack et al., 2015; Poldrack et al., 2017). Indeed, statistical simulations show that correlation measures are highly unstable at low sample sizes (Schönbrodt & Perugini, 2013). Here, we had a sample size of 30 participants, which was the same as in the first of the previously cited work by Schwarzkopf et al. (2011) and larger than in the other studies mentioned (*N *= 26 [Schwarzkopf & Rees, 2013]); *N* = 20 [Song et al., 2013]; and *N *= 10 [Moutsiana et al., 2016]). Although our sample constitutes an above-average sample size in neuroimaging studies, it is still not sufficient to detect small effects. Schwarzkopf et al. (2011) reported medium correlations between V1 surface area and EB illusion (*r* = −0.38) and PZ illusion (*r* = −0.48) magnitudes, which, assuming a significance level of *α* = 0.05, corresponds with an achieved power of 0.560 and 0.790, respectively. Notably, correlations of less than 0.37 will not reach statistical significance with a sample size of 30 and explain less than 14% of the variance. Furthermore, in the work of Moutsiana et al. (2016), the sample size was 10, but the authors treated the 4 dependent data points from the 4 visual quadrants of each participant as independent data in their statistical analyses, ending up with an artificially inflated sample size of 40. These sampling issues could partially explain our failure to replicate the previous findings.

To summarize, our results speak against the existence of neural common factors for visual illusions, at least in terms of simple neural measures such as pRF size or early visual surface area. Our study goes beyond a failure to replicate, showing that the deeply engrained view about common factors does not seem to hold for visual illusions. The great variability in both neural and perceptual measures, with low correlations at the perceptual level, makes it unlikely that there are simple links between variability on one and the other level (Ozkirli, Herzog, & Jastrzębowska, 2025). We find it important to highlight the persisting bias against publishing null results and replication failures, which contributes to an inflated perception of simple brain–behavior relationships.

## Conclusions

Considering the highly complex and recurrent nature of visual processing, we advocate for the need to shift studies of interindividual variability to more sophisticated neuroimaging data acquisition and analysis methods, for example, predicting the pattern of visual illusion results based on patterns in brain data (Charest & Kriegeskorte, 2015).

## Supplementary Material

Supplement 1
