# Supplementary material for: Is there a neural common factor for visual illusions?
Source: J Vis. 2026 Oct 13;26(10):13. doi: 10.1167/jov.26.10.13 (PMC13676236; doi:10.1167/jov.26.10.13)
Supplement: Supplementary file 1 [file jovi-26-10-13_s001.pdf]

Supplementary Information. Is there a neural common factor for visual illusions?

Maya A. Jastrzębowska[a, b, c *], Ayberk Ozkirli[a, b, d], Aline F. Cretenoud[a], Bogdan Draganski[b, e, f], Michael H. Herzog[a]

[a] *Laboratory of Psychophysics, Brain Mind Institute, School of Life Sciences, École Polytechnique Fédérale de Lausanne (EPFL), Switzerland*

[b] *Laboratory for Research in Neuroimaging (LREN), Centre for Research in Neurosciences, Department of Clinical Neurosciences, Lausanne University Hospital and University of Lausanne, Switzerland*

[c] *Department of Psychology, University of Warsaw, Poland*

[d] *Neuro-X Institute, School of Engineering, École Polytechnique Fédérale de Lausanne (EPFL), Geneva, Switzerland*

[e] *Imaging Neuroscience of Ageing – inAGE laboratory, Neurology Department-Inselspital, University Hospital Bern, University Bern, Bern, Switzerland*

[f] *Institute for diagnostic and interventional neuroradiology, Inselspital Bern, University of Bern, Switzerland*

* corresponding author: maya.jastrzebowska@gmail.com

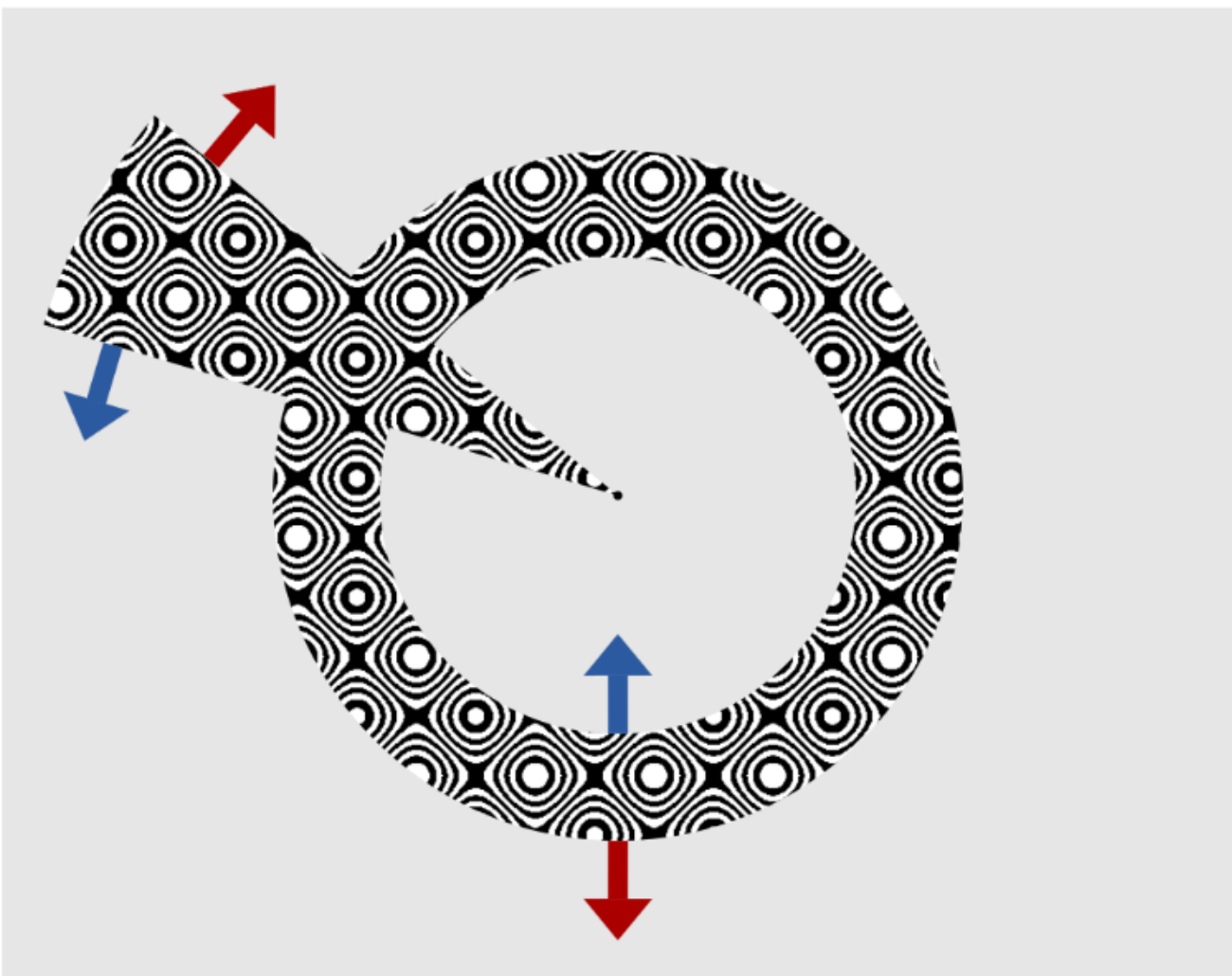

Figure S1. PRF mapping stimulus. The stimulus consisted of a rotating wedge and an expanding and contracting ring aperture, which reveals a ripple texture background. Six sessions of pRF mapping were included in the experiment. In each session, the wedge completed three cycles and the ring completed five cycles. During odd-numbered sessions, the wedge rotated clockwise, and the ring started out by expanding (indicated by red arrows). During even-numbered sessions, the wedge rotated counterclockwise, and the ring started out by contracting (indicated by blue arrows).

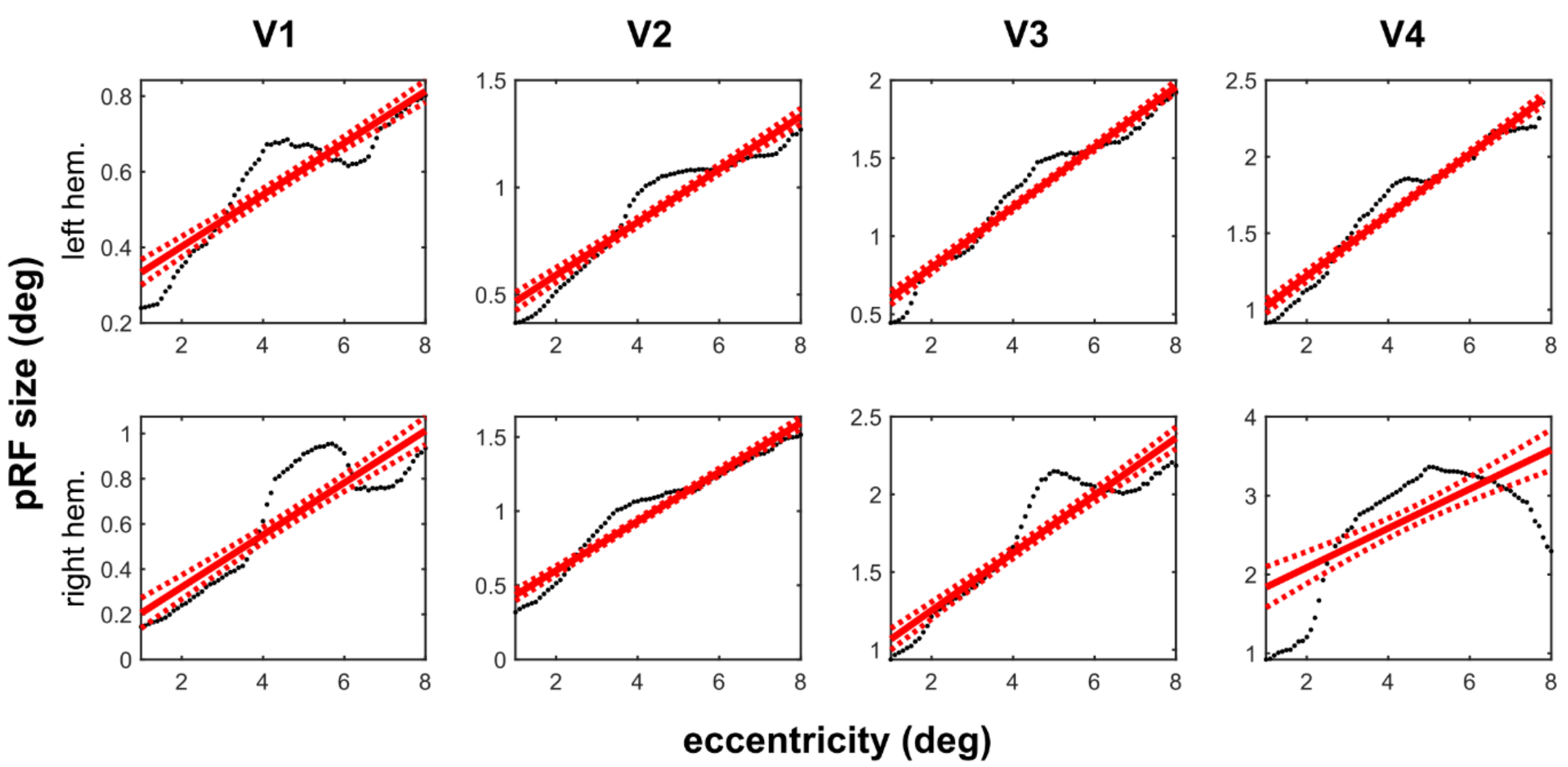

Figure S2. Linear regression model fit to pRF size as a function of eccentricity, weighted by the pRF model's goodness-of-fit (R^2), across ROIs and in the two hemispheres for a sample participant. All slopes are positive, as would be expected based on known properties of retinotopic organization. Dotted red lines represent the 95% confidence bounds.

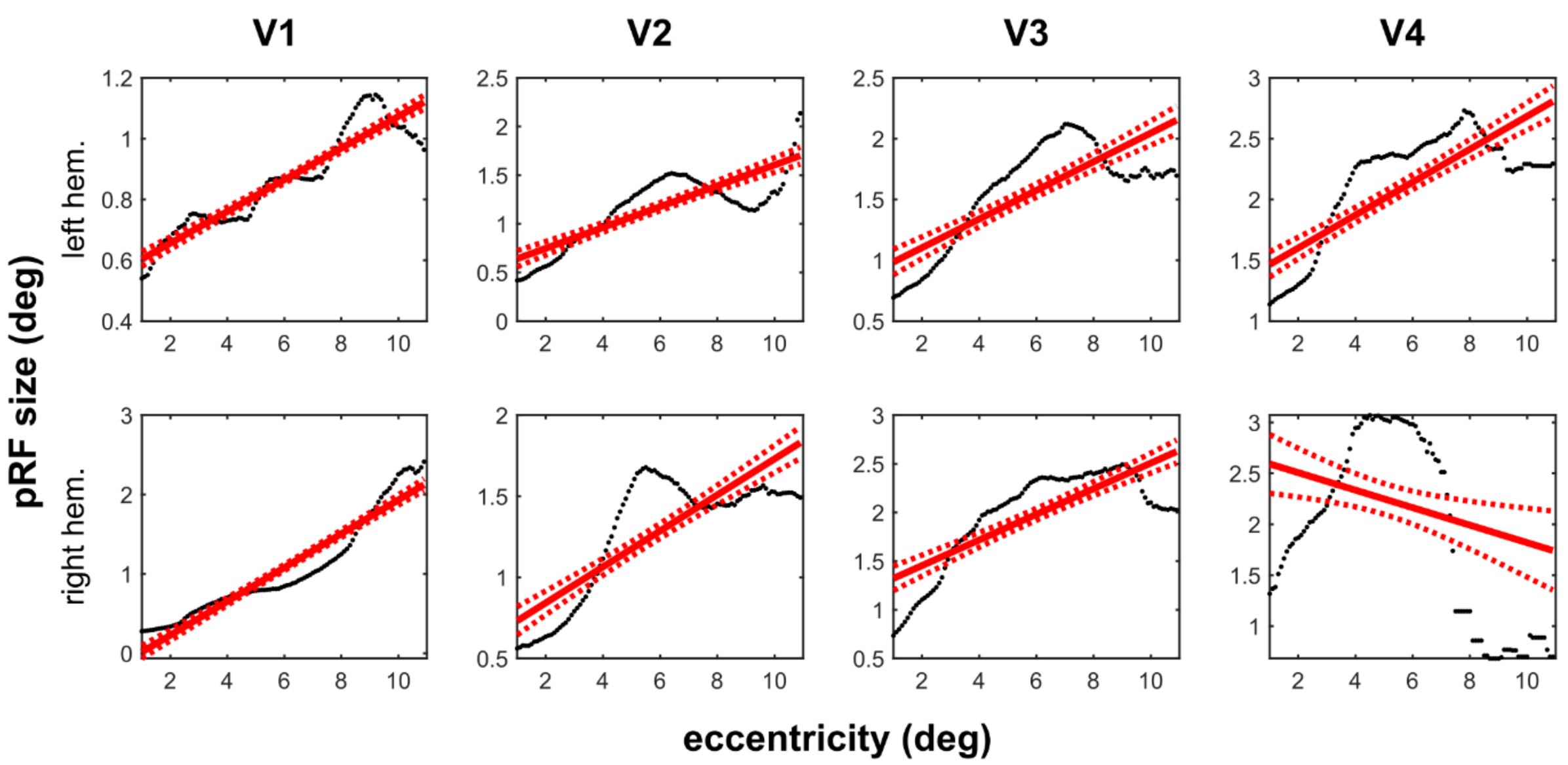

Figure S3. Linear regression model fit to pRF size as a function of eccentricity, weighted by the pRF model's goodness-of-fit (R^2), across ROIs and in the two hemispheres for another sample participant. Here, due to noisy pRF estimates in higher eccentricities, slope is estimated to be negative in V4 of the right hemisphere. Such data points were discarded. Dotted red lines represent the 95% confidence bounds.

Table S1. Intra-rater reliabilities expressed as intraclass coefficients (ICC) for both reference-dependent conditions of each illusion (N = 30). *P*-values were not corrected for multiple comparison (i.e., $\alpha = 0.05$).

Illusion	Condition	ICC coef.	95% CI	*df*	*p*
BS	horizontal	0.622	[0.394, 0.777]	[29, 29]	<0.001
	vertical	0.540	[0.285, 0.723]	[29, 29]	0.001
CS	left	0.274	[-0.029, 0.531]	[29, 29]	0.068
	right	0.149	[-0.159, 0.430]	[29, 29]	0.212
DB	left	0.478	[0.207, 0.681]	[29, 29]	0.003
	right	0.597	[0.360, 0.761]	[29, 29]	<0.001
EB1	small	0.784	[0.632, 0.878]	[29, 29]	<0.001
	large	0.304	[0.004, 0.554]	[29, 29]	0.048
EB2	small	0.608	[0.376, 0.769]	[29, 29]	<0.001
	large	0.686	[0.486, 0.818]	[29, 29]	<0.001
EX	black	0.763	[0.599, 0.865]	[29, 29]	<0.001
	white	0.906	[0.832, 0.948]	[29, 29]	<0.001
HC	black	0.921	[0.858, 0.957]	[29, 29]	<0.001
	white	0.840	[0.721, 0.911]	[29, 29]	<0.001
ML	outward	0.528	[0.270, 0.715]	[29, 29]	0.001
	inward	0.658	[0.445, 0.800]	[29, 29]	<0.001
PD	left	0.394	[0.105, 0.621]	[29, 29]	0.014
	right	0.699	[0.505, 0.826]	[29, 29]	<0.001
PZ	down	0.713	[0.525, 0.835]	[29, 29]	<0.001
	up	0.654	[0.440, 0.798]	[29, 29]	<0.001
PZh	down	0.850	[0.737, 0.916]	[29, 29]	<0.001
	up	0.642	[0.422, 0.790]	[29, 29]	<0.001
TT	left	0.340	[0.044, 0.581]	[29, 29]	0.031
	right	0.511	[0.249, 0.704]	[29, 29]	0.002
ZN	left	0.534	[0.278, 0.720]	[29, 29]	0.001
	right	0.773	[0.615, 0.871]	[29, 29]	<0.001

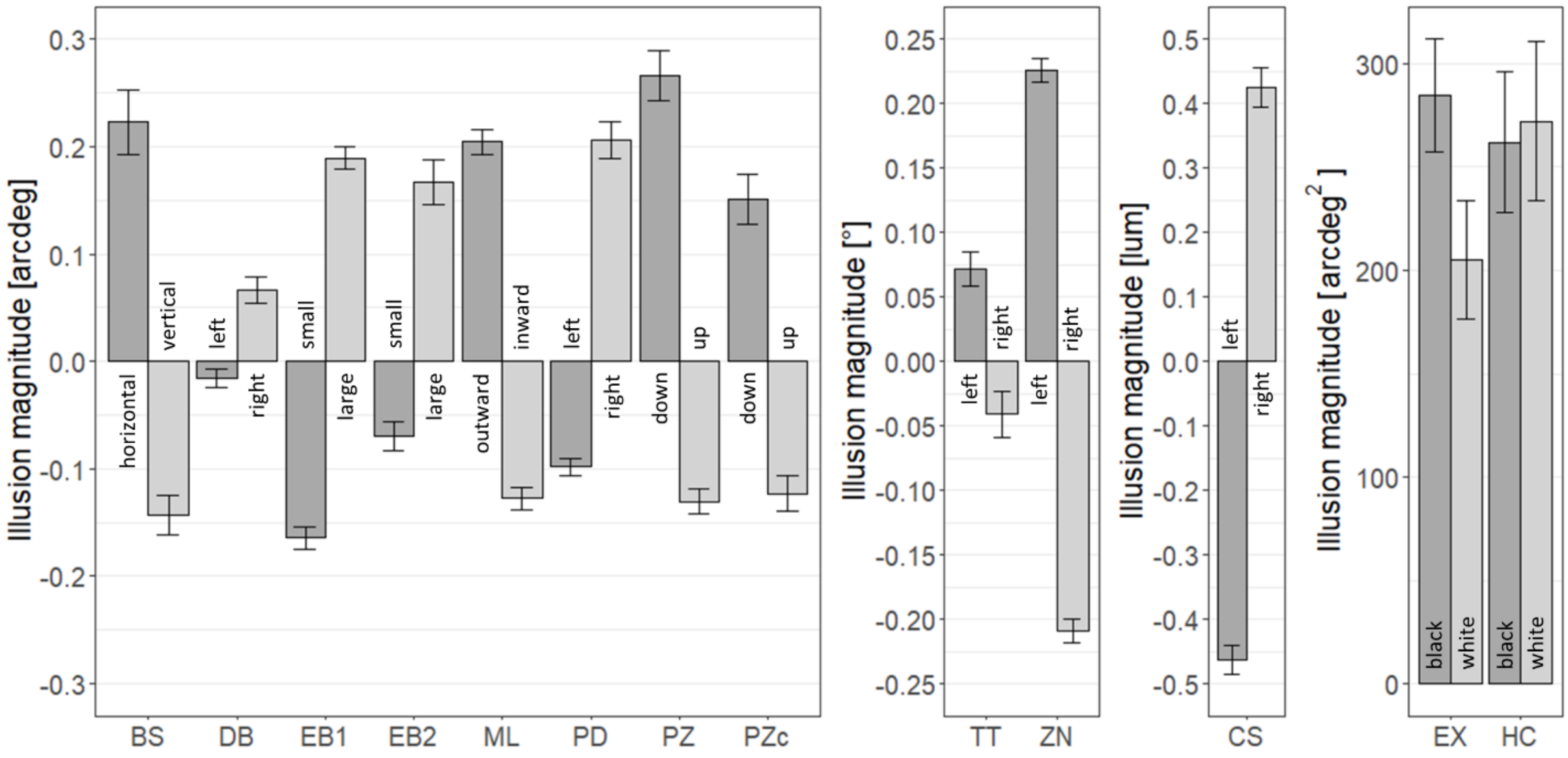

Figure S4. Illusion magnitudes for both reference-dependent conditions of each illusion. For all but the EX and HC illusions, positive and negative magnitudes indicate over- and under-adjustments compared to the reference, respectively. Error bars correspond to the standard error of the mean (SEM).

Table S2. Illusion magnitudes are expressed as a difference compared to the reference, except in the EX and HC conditions, where we extracted the area in which all shapes were perceptible to the participants. Illusion magnitudes are summarized as mean (*M*), standard deviation (*SD*), standard error of the mean (*SEM*), and 95% confidence interval (95% *CI*) for both reference-dependent conditions of each illusion (N = 30).

Illusion	Condition	*M*	*SD*	*SEM*	95% *CI*	Unit
BS	horizontal	0.223	0.163	0.030	0.061	arcdeg
	vertical	-0.143	0.101	0.018	0.038	
CS	left	-0.462	0.123	0.022	0.046	luminance
	right	0.425	0.166	0.030	0.062	
DB	left	-0.015	0.047	0.009	0.017	arcdeg
	right	0.066	0.067	0.012	0.025	
EB1	small	-0.164	0.058	0.011	0.022	arcdeg
	large	0.189	0.056	0.010	0.021	
EB2	small	-0.070	0.074	0.013	0.027	arcdeg
	large	0.167	0.114	0.021	0.043	
EX	black	284.659	148.808	27.169	55.566	$arcdeg^2$
	white	204.984	158.165	28.877	59.060	
HC	black	261.930	186.635	34.075	69.691	$arcdeg^2$
	white	272.082	210.487	38.429	78.597	
ML	outward	0.205	0.064	0.012	0.024	arcdeg
	inward	-0.128	0.057	0.010	0.021	
PD	left	-0.098	0.046	0.008	0.017	arcdeg
	right	0.206	0.093	0.017	0.035	
PZ	down	0.266	0.126	0.023	0.047	arcdeg
	up	-0.130	0.064	0.012	0.024	
PZh	down	0.151	0.127	0.023	0.048	arcdeg
	up	-0.123	0.091	0.017	0.034	
TT	left	0.072	0.071	0.013	0.027	°
	right	-0.041	0.098	0.018	0.037	
ZN	left	0.226	0.050	0.009	0.019	°
	right	-0.209	0.050	0.009	0.019	

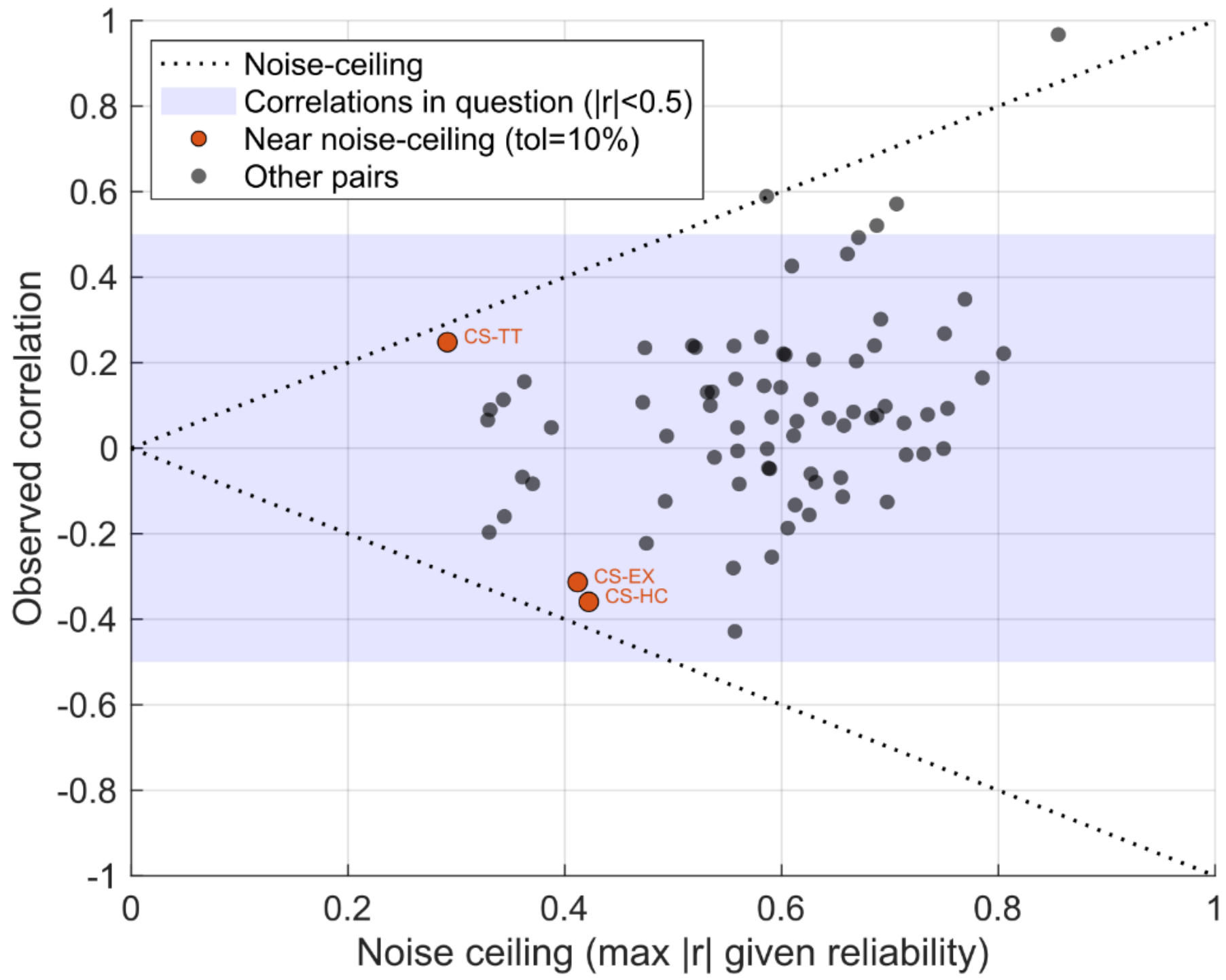

Figure S5. Observed between-illusion correlations relative to noise ceiling. Only three of the low-to-medium ($|r| < 0.5$) between-illusion correlations (CS-TT, CS-EX, CS-HC) lay within 10% of the noise ceiling. These exceptions were driven by the relatively low reliability of the contrast (CS) illusion.

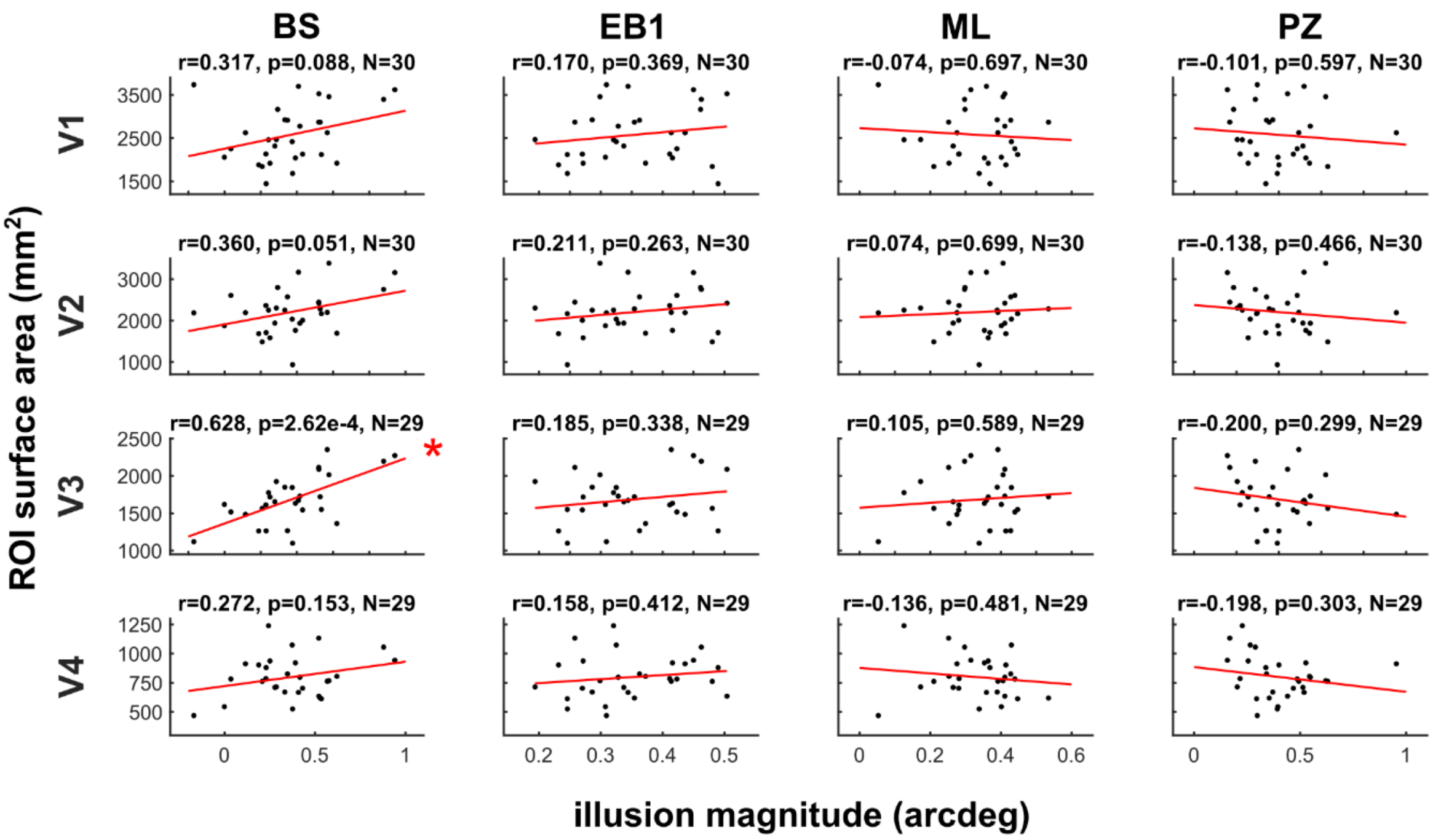

Figure S6. Correlations between ROI surface area and visual illusion magnitudes for other size illusions. BS – bisection, EB1 – Ebbinghaus variant 1, ML – Müller-Lyer, PZ – Ponzo. Asterisks mark correlations that are significant ($p < 0.05$, uncorrected). Outliers (modified z-score > 3.5) removed. All illusion magnitudes are expressed in units of arcdeg.

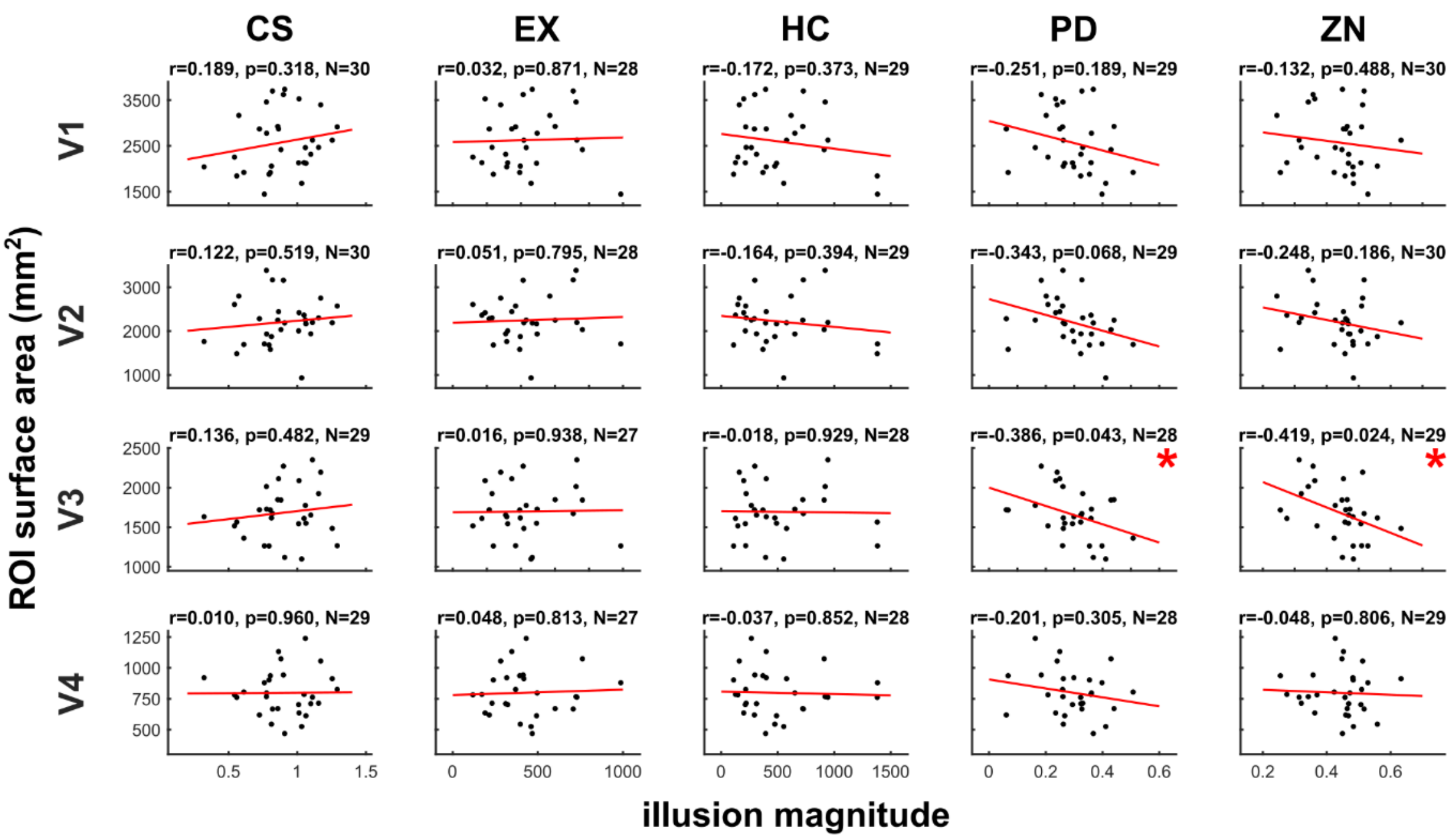

Figure S7. Correlations between ROI surface area and visual illusion magnitudes for remaining illusions (uniform textures, contrast, perceived orientation). CS – contrast, EX – Extinction, HC – Honeycomb, PD – Poggendorff, ZN – Zoellner. Asterisks mark significant correlations (p<0.05, uncorrected). Outliers (modified z-score > 3.5) removed. Illusion magnitudes are expressed in different units for different illusions (see **Table S2**).

Table S3. Results of Bayesian linear regression analysis of illusion magnitudes (dependent variable), using slope and intercept of pRF size as a function of eccentricity as covariates. Results consist of Bayes Factors (BF_{10}) of three alternative models w.r.t. the null model: (1) model that includes the slope, (2) model that includes the intercept, (3) model that includes both slope and intercept. Instances of substantial evidence in favor of either the null or the alternative model (BF ∈ [3,10) are marked in bold with an asterisk. Instances of substantial evidence in favor of the null hypothesis (BF_{10} ∈ [0.1,0.333), corresponding to BF_{01} ∈ [3,10)) are marked in red and bold with a dagger (†). Outliers (modified z-score > 3.5) removed.

Visual area	Illusion	N	BF_{10}		
			slope	intercept	slope+intercept
V1	BS	30	0.384	0.404	0.334
	CS	30	0.359	0.528	**0.260†**
	DB	30	0.545	1.377	0.580
	EB1	30	0.354	0.346	**0.168†**
	EB2	29	0.515	0.380	**0.246†**
	EX	28	0.916	0.792	**0.103†**
	HC	29	0.765	0.373	0.386
	ML	30	0.442	0.406	**0.208†**
	PD	29	0.464	0.481	0.991
	PZ	30	0.349	0.516	**0.270†**
	PZh	28	1.161	0.653	0.507
	TT	30	0.398	0.517	0.706
	ZN	30	0.487	0.345	**0.301†**
V2	BS	30	0.901	0.368	0.517
	CS	30	0.526	0.352	**0.276†**
	DB	30	0.431	0.880	0.403
	EB1	30	0.356	0.344	**0.174†**
	EB2	29	0.350	0.402	**0.234†**
	EX	28	0.476	0.356	0.337
	HC	29	0.640	0.356	0.639
	ML	30	0.360	0.351	**0.171†**
	PD	29	0.370	1.082	2.523
	PZ	30	0.366	0.350	**0.201†**
	PZh	28	0.428	0.379	**0.205†**
	TT	30	0.663	0.363	0.916
	ZN	30	0.350	0.415	**0.262†**
V3	BS	30	0.677	1.565	0.669
	CS	30	0.358	0.395	**0.332†**
	DB	30	0.572	1.984	0.998
	EB1	30	0.351	0.345	**0.169†**
	EB2	29	0.350	0.488	0.430
	EX	28	0.353	0.354	**0.174†**
	HC	29	0.355	0.349	**0.182†**
	ML	30	0.347	0.585	0.469
	PD	29	2.331	1.853	1.074
	PZ	30	0.358	0.371	**0.176†**
	PZh	28	0.378	0.361	**0.184†**
	TT	30	0.359	0.364	**0.173†**
	ZN	30	0.408	0.411	**0.196†**
V4	BS	30	0.471	0.344	**0.307†**
	CS	30	0.356	0.525	**0.274†**
	DB	30	0.412	0.368	**0.194†**
	EB1	30	0.387	0.393	0.365
	EB2	29	0.782	0.348	0.809
	EX	28	0.359	0.438	**0.225†**

	HC	29	0.352	0.388	**0.228†**
	ML	30	0.940	0.650	0.416
	PD	29	0.348	0.349	**0.168†**
	PZ	30	0.344	0.415	**0.228†**
	PZh	28	0.689	0.370	0.395
	TT	30	0.344	0.350	**0.168†**
	ZN	30	1.016	0.918	0.501
